# Supplementary material for: Time-aggregated mobile phone mobility data are sufficient for modelling influenza spread: the case of Bangladesh
Source: J R Soc Interface. 2020 Jun 17;17(167):20190809. doi: 10.1098/rsif.2019.0809 (PMC7328378; doi:10.1098/rsif.2019.0809)
Supplement: Electronic supplementary information for Time-aggregated mobile phone mobility data are sufficient for modelling influenza spread: the case of Bangladesh [file rsif20190809supp1.pdf]

Electronic supplementary information for Time-aggregated mobile phone mobility data are sufficient for modelling influenza spread: the case of Bangladesh

Solveig Engebretsen[1,2,3], Kenth Engø-Monsen[4], Mohammad Abdul Aleem[5], Emily Suzanne Gurley[5,6], Arnoldo Frigessi[1,7], Birgitte Freiesleben de Blasio[1,2]

[1] *Oslo Centre for Biostatistics and Epidemiology, University of Oslo*
[2] *Department of Method Development and Analytics, Norwegian Institute of Public Health*
[3] *Norwegian Computing Center*
[4] *Telenor Research, Oslo, Norway*
[5] *International Centre for Diarrhoeal Disease Research, Bangladesh, ICDDR,B*
[6] *Department of Epidemiology, Johns Hopkins Bloomberg School of Public Health*
[7] *Oslo Centre for Biostatistics and Epidemiology, Oslo University Hospital*

Manuscript accepted in the Journal of the Royal Society Interface.

S1 Incompleteness of mobile phone mobility data

The mobility data is completely lacking for five upazilas of Bangladesh in the Chittagong division, and our model thus does not include these upazilas. These upazilas are Thanchi, Barkal, Belai Chhari, Jurai Chhari, and Langadu. The transitions to and from the upazila Parshuram (Chittagong division) are missing, but the total count of active mobile phones in Parshuram is observed, allowing us to estimate the population size in Parshuram. We use the gravity model to estimate the transitions to and from Parshuram.

S2 Descriptive statistics of the hospital sentinel case data

Descriptives for the hospital sentinel cases for the different seasons are given in Table S1. Note that both influenza B and influenza A types were circulating in 2014, 2016, and 2017, while 2015 was mainly dominated by A(H1N1). Most of the type A cases in 2014 and 2016 were of subtype A(H3).

Table S1: **Case data descriptives.**

						Influenza (strain)				
Year	Tested	Positive	Mean age (sd)	Male	Female	A	A(H1N1)	A(H3)	B	A+B
2014	3826	590	30.0 (23.6)	348	242	312	6	304	277	1
2015	3567	508	27.3 (22.4)	302	206	476	380	94	32	0
2016	4276	687	27.8 (23.4)	400	287	298	6	291	385	4
2017	4229	890	26.5 (22.4)	503	387	657	383	273	232	1

Descriptive statistics for the hospital sentinel influenza case data for 2014, 2015, 2016, and 2017.

S3 Infectious disease model description

The influenza dynamics model is a stochastic compartmental $SEII_aR$ metapopulation model, similar to the model previously published in (Engebretsen et al., 2019). Here the compartments are the individuals who are susceptible to the disease (S), the exposed individuals (E), that is, those who have been infected but are not yet infectious, the symptomatic infectious individuals (I), the asymptomatic infectious individuals (I_a), and the recovered/removed individuals (R). See also Balcan et al. (2009) and Colizza et al. (2007). We let β denote the transmission probability per unit time, $1/\lambda$ be the average latent period, $1/\gamma$ be the average infectious period, and p_a denote the probability that an infection is asymptomatic. We assume that the transmission probability of the asymptomatic infectious is reduced by r_β. The stochastic $SEII_aR$ equations are given by:

$$S^i(t+\Delta t) = S^i(t) - X_1^i(t),$$

$$E^i(t+\Delta t) = E^i(t) + X_1^i(t) - X_2^i(t) - X_3^i(t),$$

$$I^i(t+\Delta t) = I^i(t) + X_2^i(t) - X_4^i(t),$$

$$I_a^i(t+\Delta t) = I_a^i(t) + X_3^i(t) - X_5^i(t),$$

where $S^i(t)$, $E^i(t)$, $I^i(t)$, $I_a^i(t)$, and N_i^t are the number of susceptible individuals, exposed individuals, symptomatic infectious individuals, asymptomatic infectious individuals, and the total population at time t in location i, respectively. $X_1^i(t) \sim \text{Binom}\left(S^i(t),\ \beta\Delta t I^i(t)/N_i^t + r_\beta\beta\Delta t I_a^i(t)/N_i^t\right)$, $X_2^i(t), X_3^i(t) \sim \text{Multinom}\left(E^i(t),\ (1-p_a)\lambda\Delta t,\ p_a\lambda\Delta t\right)$, $X_4^i(t) \sim \text{Binom}\left(I^i(t),\ \gamma\Delta t\right)$, $X_5^i(t) \sim \text{Binom}\left(I_a^i(t),\ \gamma\Delta t\right)$, $\text{Binom}(n,p)$ is the binomial distribution with n trials and success probability p, and $\text{Multinom}(n,p_1,p_2)$ is the multinomial distribution with n trials and success probabilities p_1 and p_2. We assume that the population size is constant during the epidemic, so that $R = N - S - E - I - I_a$. As in Balcan et al. (2009), we fix the probability of an infectious individual being asymptomatic to $p_a = 0.33$, similar to the estimated asymptomatic proportion in Presanis et al. (2011); Birrell et al. (2011). We let the transmission probability of the asymptomatic infectious be reduced by 50%, so we fix $r_\beta = 0.50$, as in Balcan et al. (2009). The basic reproductive number R_0 is defined as the number of expected new cases generated by one infectious individual, in a fully susceptible population. The basic reproductive number of the model is given by $R_0 = \frac{\beta}{\gamma}(0.5 \cdot 0.33 + 0.67)$ (Colizza et al., 2007). As we cannot assume that the population is fully susceptible, we will estimate the effective reproductive number, R_e, which takes the prior immunity into account.

S4 Prior distributions

S4.1 Prior distribution and parameter specifications

We assume prior distributions for the transmission parameter β and the reporting probability r. It is reasonable to assume that only a small proportion of the cases are reported. Some individuals do not go to their doctor with influenza. Moreover, since we only have information for some hospitals, it should be a small fraction of the total number of influenza cases. We assume a beta distribution for r, with shape parameters 1 and 7, corresponding to a mean 1/8 and variance $7/(8^2 \cdot 9)$. This prior is quite wide and uninformative, but skewed towards lower reporting probabilities. A plot of the prior distribution for the reporting rate is provided in Figure S1. For the transmission parameter β, we assume a uniform prior on $(0.4, 0.7)$, which is quite uninformative. When we estimate the parameters for the other seasons than 2017, we use the results for the 2017 season to define our priors.

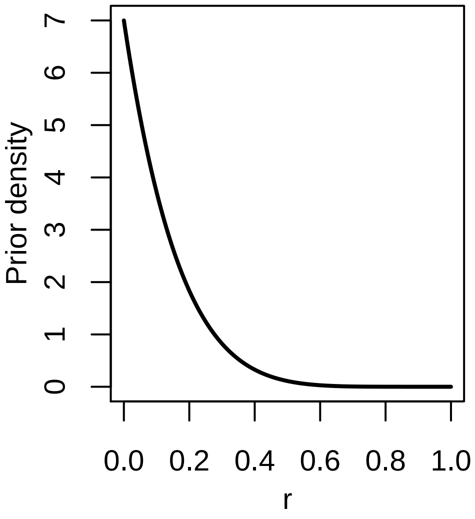

Figure S1: Prior distribution for the reporting probability r.

We fix the mean latent period and the mean infectious period. The mean latent period is fixed to 1.9 days, as in Balcan et al. (2009) and Longini Jr et al. (2004). This is also in accordance with the fact sheet from WHO on seasonal influenza (World Health Organization, 2016), where the average incubation period is claimed to be two days. The mean infectious period is fixed to three days. Similar infectious and/or latent periods for seasonal and pandemic influenza can also be found in the literature in Corbella et al. (2018); Birrell et al. (2017); Balcan et al. (2009); Colizza et al. (2007); Germann et al. (2006); Longini Jr et al. (2004); Bajardi et al. (2011).

The mean latent and infectious periods are fixed, as we do not have data to inform these– Corbella et al. (2018) argue that the latent and infectious periods can only be inferred from detailed information at the individual level, which we do not have. Even though there is good knowledge of the latent and infectious period distributions in the literature, this is a strong assumption. In addition to scarce data, the different model parameters are strongly correlated, further complicating the parameter estimation. The data contain little or no information on the latent period, and the infectious period is correlated with both the transmission parameter and the reporting probability.

S5 Posterior distribution

A scatterplot of the joint posterior for the 2017 season is given in Figure S2. The posterior correlation is -0.0525.

S6 ABC-SMC procedure for inference

The idea behind approximate Bayesian computation (ABC) is to use simulations from the model, and accept parameters if the simulations are sufficiently close to the observed data, measured in summary statistics. We use a sequential Monte Carlo version of ABC. We use four summary statistics–the global observed incidence curve, the observed peak height (that is, the maximum number of daily observed new infectious cases), the duration of the epidemic, and the final size of the epidemic. We define the duration as the number of days between the day with 10% of the total number of observed cases, and the day with 90% of the total number of observed cases. The final size is the total number of observed symptomatic cases. The time-averaged mobility is used in the fitting procedure, as this is computationally advantageous, and since we do not

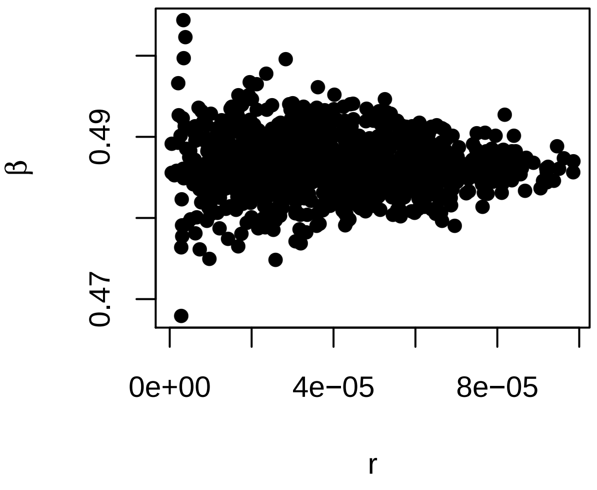

Figure S2: Scatterplot of posterior distribution for the reporting rate and the transmission rate β for 2017.

know the initial date.

We use an algorithm similar to the one in Brooks-Pollock et al. (2014), provided in Algorithm 1. In the algorithm, m is the number of summary statistics, and they are denoted by $S_1, \ldots, S_m$. The distance in the summary statistics are weighted, in order to make sure that they are on the same scale. We denote the data by D_{lt}, which is the number of observed cases in location l on day t, and the simulated observed incidence by D'_{lt}. In the proposal distribution, we use a variance which is twice the empirical variance of the previous round, as suggested in Beaumont et al. (2009). We use a Euclidean distance metric between the number of reported cases and the simulated number of observed infectious symptomatic cases, for the global incidence curves. In the comparison, we align the global peak date of the simulated data with the global peak date in the observed data. Hence, we compare the shapes of the curves. Let L be the number of locations and T the duration of the epidemic in number of days. The distance in the shape of curve statistic is thus given by $\sqrt{\sum_{t=1}^{T}(\sum_{l=1}^{L} D'_{lt} - D_{lt})^2}$. For the other summaries, duration, final size, and peak height, we use the absolute distance.

S7 Seeding locations

When investigating the spatial spread for various hypothetical seeding scenarios, we seed in Dhaka (seeding in Sutrapur upazila), and in the divisions located in the different "corners" of the country, that is, North-West (Rangpur division, seeding in Rangpur Sadar), North-East (Sylhet division, seeding in Sylhet Sadar), South-West (Khulna division, seeding in Khulna Sadar), and South-East (Chittagong division, seeding in Double Mooring). For completeness, we also include results when seeding in the remaining two divisions Barisal (seeding in Barisal Sadar), and Rajshahi (seeding in Matihar), provided in the Section S13 in this supplement.

When we simulate the 2014-2016 influenza seasons, we seed in the 11 upazilas with the earliest cases for the year in question, similar to the seeding scenario for 2017. For 2016, this corresponds to six upazilas in Dhaka, four in Chittagong, and one in Sylhet. For 2015, this

Algorithm 1 ABC-SMC

Initialise:

Set a starting value ϵ_0 for the tolerance, $r = 1$ and $\mathbf{w}^1 = (1, \ldots, 1)$.

while $\epsilon_{r-1} > \epsilon$ **do**

set $i = 0$.

while $i < 1000$ **do**

if $r = 1$ **then**

sample parameters $\boldsymbol{\theta}_i$ from the prior π.

else

Sample $\boldsymbol{\theta}^p$ from $\boldsymbol{\theta}^{r-1}$ with weights $\mathbf{w}^{r-1}$.

Propose $\boldsymbol{\theta}_i$ from an independent gaussian distribution G centered at $\boldsymbol{\theta}^p$ with variance equal to twice the empirical variance from the previous round.

If $\pi(\boldsymbol{\theta}_i) = 0$, sample a new $\boldsymbol{\theta}_p$.

end if

Run the model with $\boldsymbol{\theta}_i$, providing the simulated number of reported cases $\mathbf{D}^{'}$.

if $f(\mathbf{D}', \mathbf{D}) = ||S_1(\mathbf{D}') - S_1(\mathbf{D})|| + \ldots + |S_m(\mathbf{D}') - S_m(\mathbf{D})| < \epsilon_r$, **then**

set $\boldsymbol{\theta}_i^r = \boldsymbol{\theta}_i$, and calculate weights $\mathbf{w}_i^r$ for the parameters, as

$$\mathbf{w}_i^r = \begin{cases} 1, & \text{if } r = 0, \\ \frac{\pi(\boldsymbol{\theta_i})}{\sum_{j=1}^n w_j^{r-1} G(\boldsymbol{\theta}_i | \boldsymbol{\theta}_j^{r-1})}, & \text{otherwise,} \end{cases}$$

Increment $i = i + 1$.

end if

end while

normalise the weights as $\mathbf{w}_i^r = \frac{w_i^r}{\sum_j w_j^r}$.

set ϵ_{r+1} to the 80th percentile of $f(\mathbf{D}', \mathbf{D})$ for the accepted parameters.

Increment $r = r + 1$.

end while

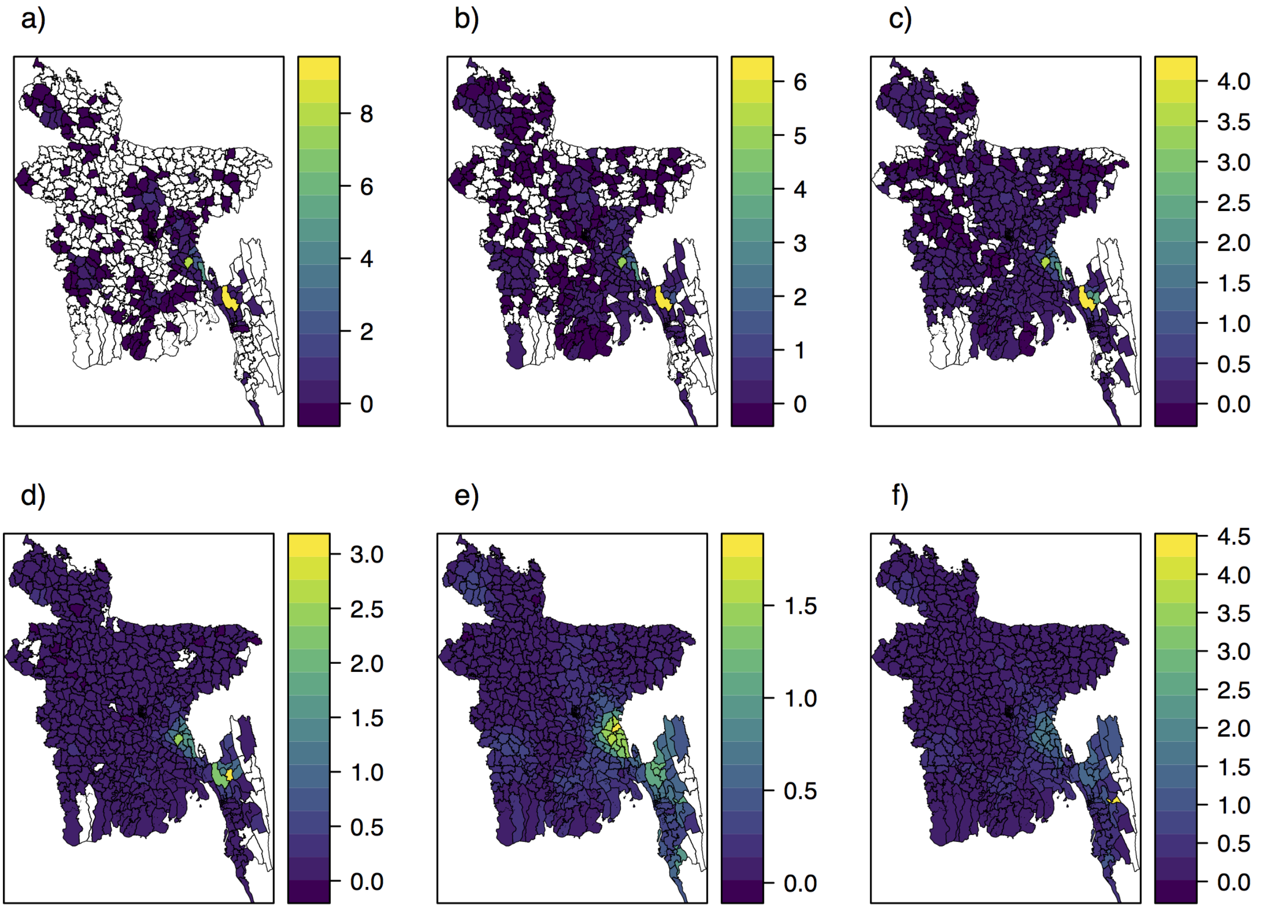

Figure S3: **Daily mobility model, 2017**. Mean prevalence per 100 000 residents in the different upazilas when seeding according to the 2017 epidemic, a) 2, b) 5, c) 10, d) 20, e) 40, and f) 50 days after the initial seeding date. Note that the scale is different for the different days.

corresponds to six upazilas in Dhaka, two in Barisal, one in Rangpur, one in Rajshahi, and one in Sylhet. For 2014, this corresponds to three upazilas in Barisal, three in Rajshahi, two in Dhaka, two in Sylhet, and one in Chittagong.

S8 Early spread for different seeding scenarios

We investigate the early spread for the different seeding settings, by plotting the mean prevalence in each upazila 2, 5, 10, 20, 40, and 50 days after the initial seeding date (day 0).

S8.1 2017 simulation

The early spatial spread for the 2017 simulation is given in Figure S3. The disease quickly spreads through the country, and most of the early spread seems to be to the neighbouring upazilas, but also to larger cities.

S8.2 Dhaka

The early spatial influenza spread when seeding in Dhaka is given in Figure S4. The disease quickly spreads to the larger cities, but also to upazilas within Dhaka division.

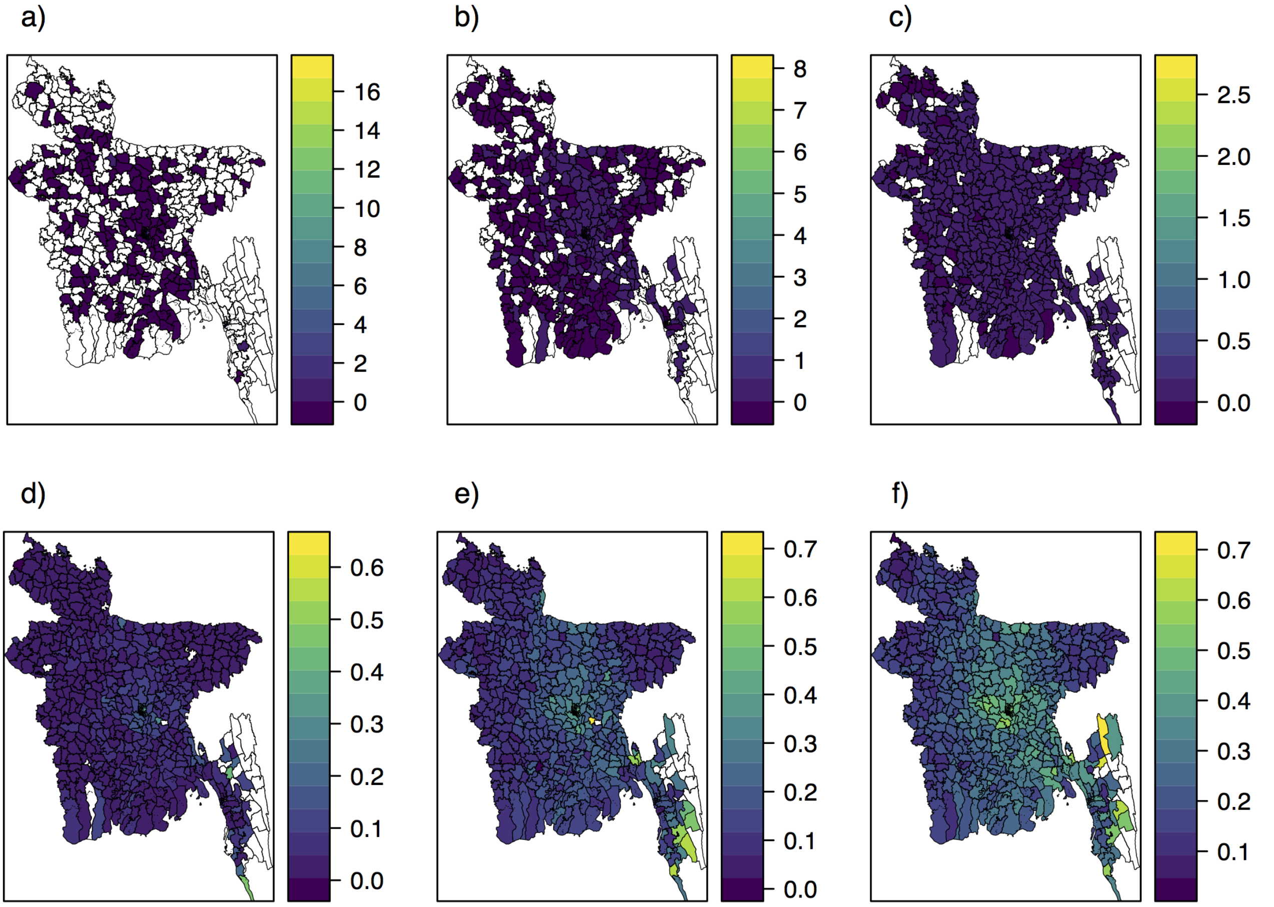

Figure S4: **Daily mobility model, Dhaka**. Mean prevalence per 100 000 residents in the different upazilas when seeding in Dhaka, on day a) 2, b) 5, c) 10, d) 20, e) 40, and f) 50. Note that the scale is different for the different days.

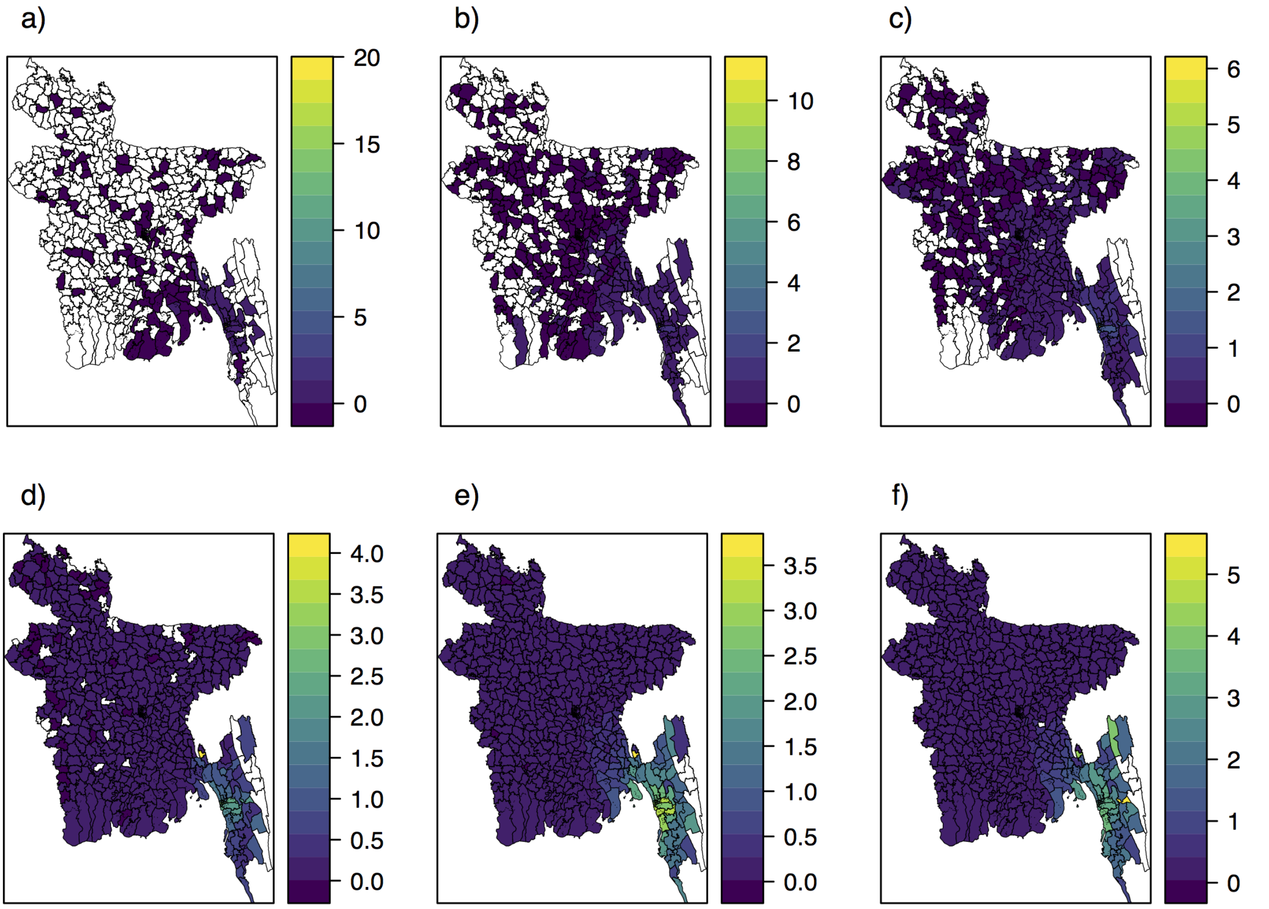

Figure S5: **Daily mobility model, Chittagong**. Mean prevalence per 100 000 residents in the different upazilas when seeding in Chittagong, a) 2, b) 5, c) 10, d) 20, e) 40, and f) 50 days after the seeding event. Note that the scale is different for the different days.

S8.3 Chittagong

The early spatial influenza spread is given in Figure S5. The disease quickly spreads to some of the larger cities (including Dhaka), but also to upazilas within Chittagong.

S8.4 Khulna

The early spatial influenza spread when seeding in Khulna is given in Figure S6. There is early spread to some of the larger cities, including Dhaka, but most of the early spread is within the Khulna division.

S8.5 Rangpur

The early spatial influenza spread when seeding in Rangpur is given in Figure S7. We see that we have early spread to Dhaka city, but that most of the early spread is in the North West.

S8.6 Sylhet

The early spatial influenza spread when seeding in Sylhet is given in Figure S8. There is early spread to Dhaka city, but most of the early spread is within the Sylhet division.

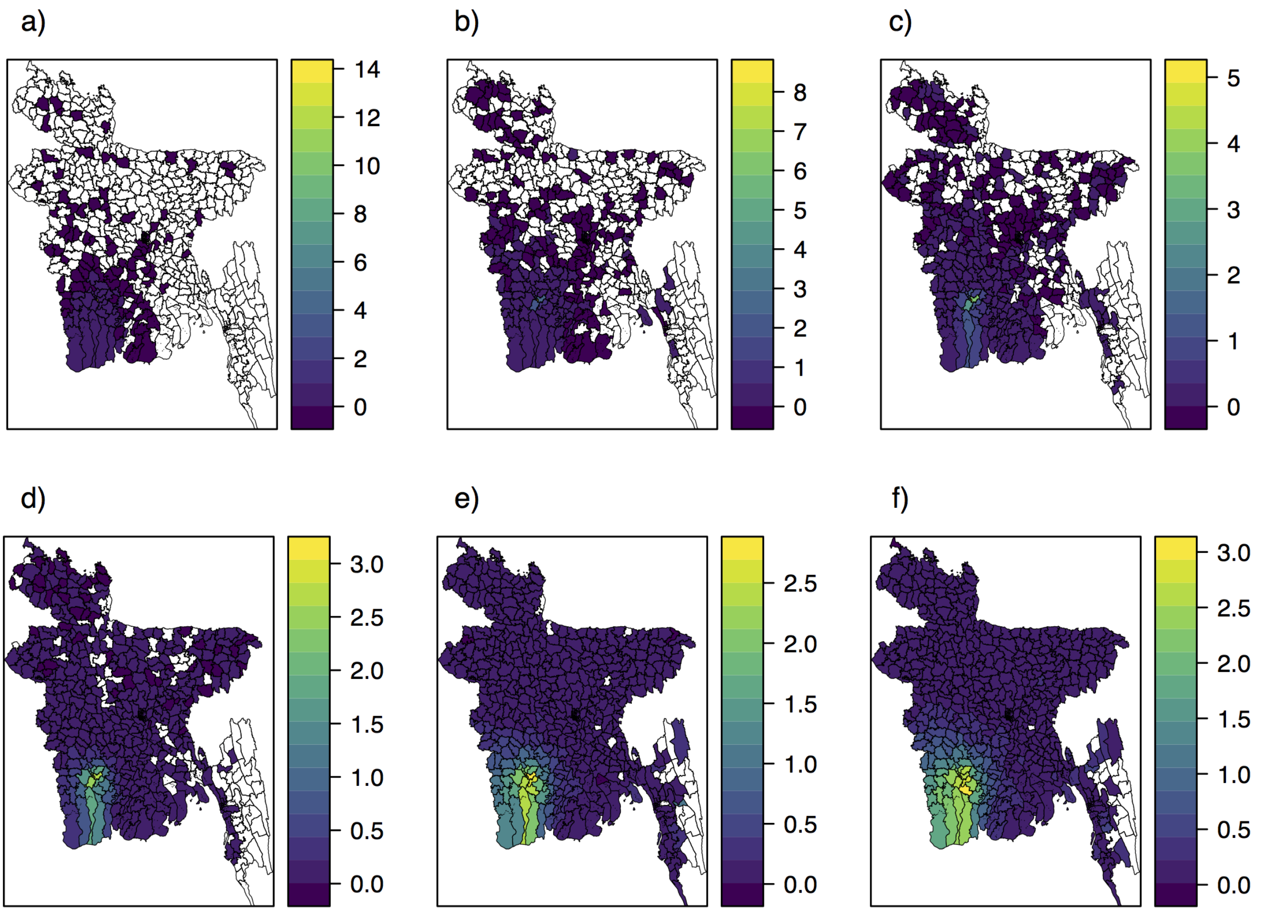

Figure S6: **Daily mobility model, Khulna**. Mean prevalence per 100 000 residents in the different upazilas when seeding in Khulna, a) 2, b) 5, c) 10, d) 20, e) 40, and f) 50 days after the seeding event. Note that the scale is different for the different days.

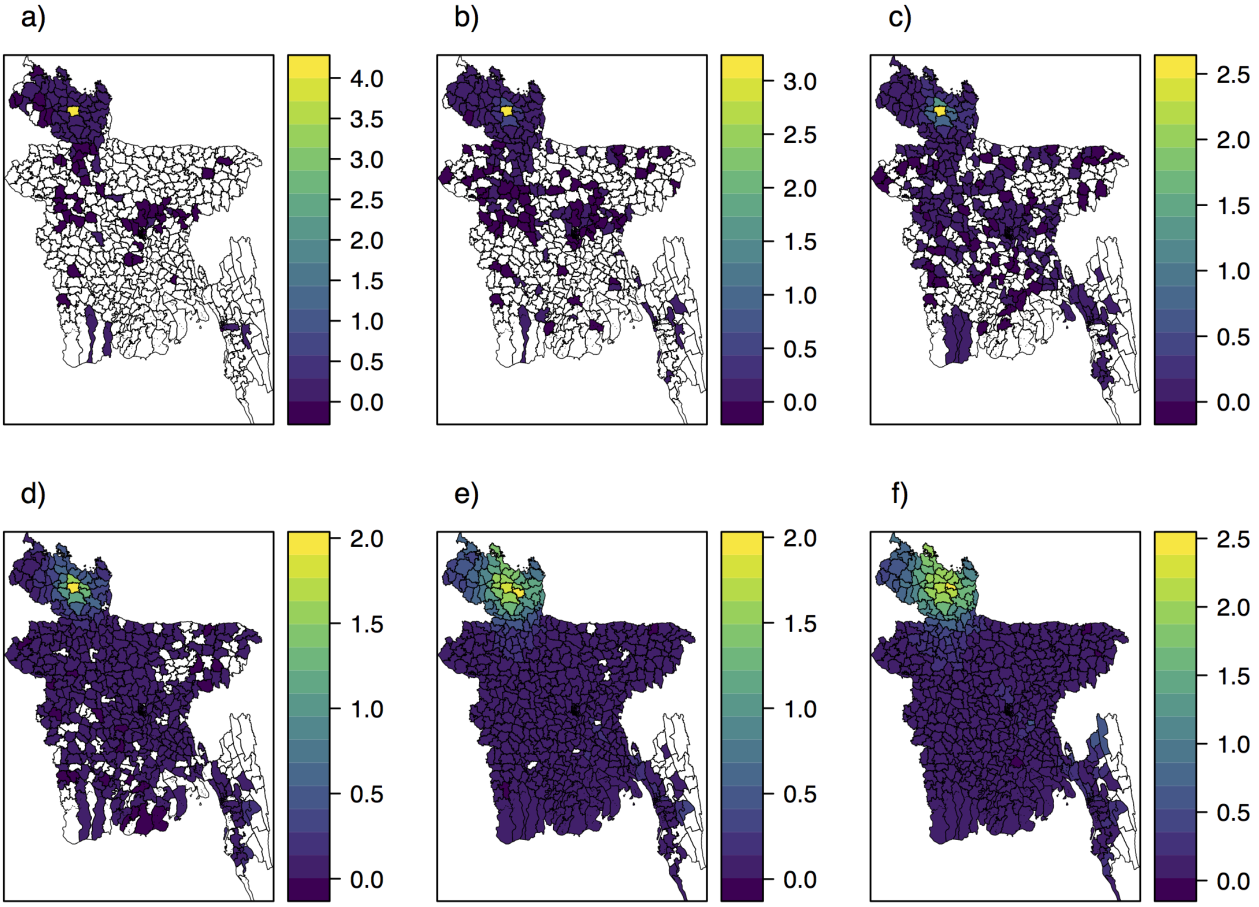

Figure S7: **Daily mobility model, Rangpur**. Mean prevalence per 100 000 residents in the different upazilas when seeding in Rangpur, a) 2, b) 5, c) 10, d) 20, e) 40, and f) 50 days after the seeding event. Note that the scale is different for the different days.

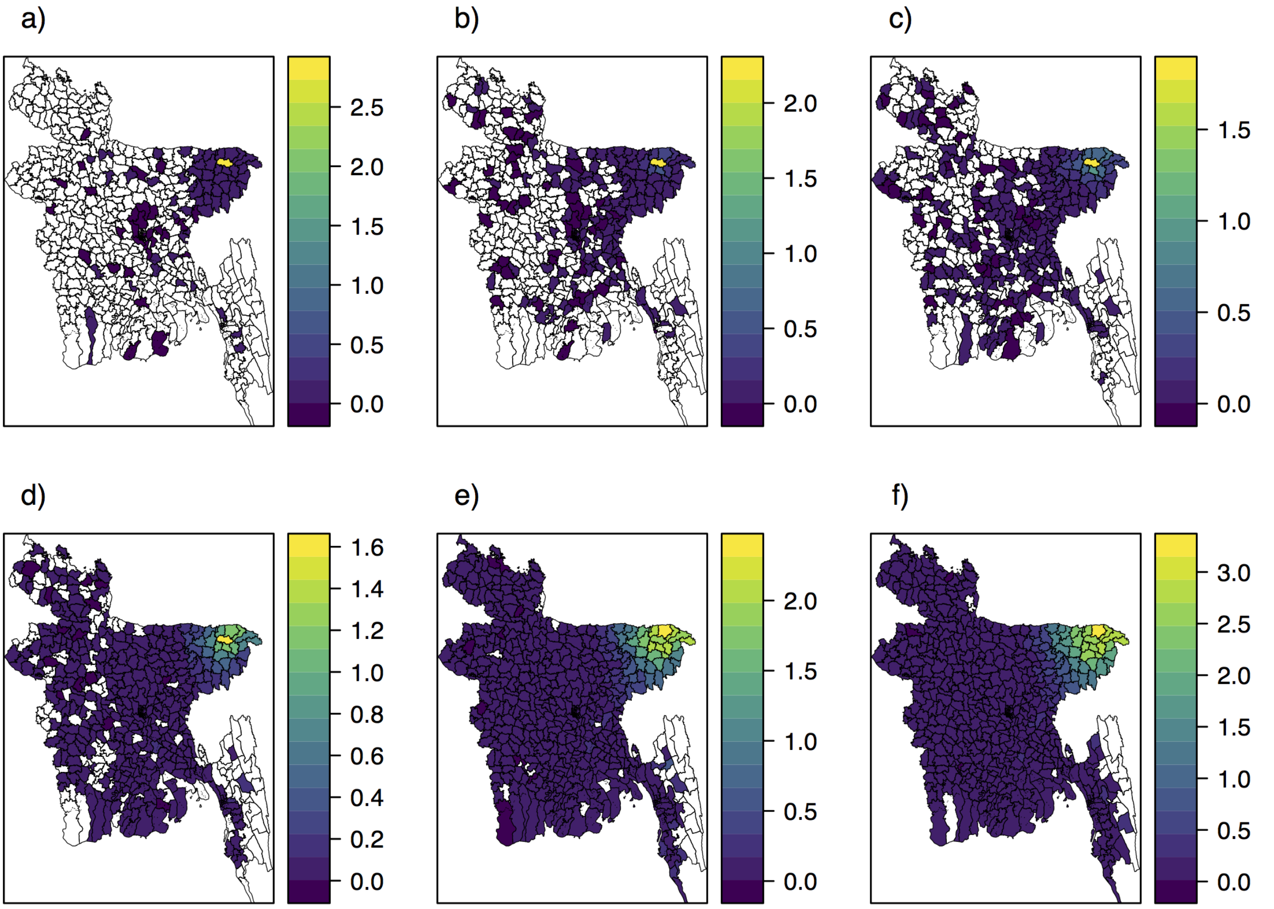

Figure S8: **Daily mobility model, Sylhet**. Mean prevalence per 100 000 residents in the different upazilas when seeding in Sylhet, a) 2, b) 5, c) 10, d) 20, e) 40, and f) 50 days after the seeding event. Note that the scale is different for the different days.

S9 Correlation between upazila initial dates

We compute the correlations between the mean initial dates in each upazila for the model using the daily mobility and the various mobility approximations. In addition, we compute the correlation between the model with the time-averaged mobility and the model with the daily mobility, when seeding on the 1st of April 2017. The correlations are provided in Table S2. The time-averaged mobility has a much higher correlation with the daily mobility than the gravity model and the radiation model.

Table S2: **Correlation of initial dates.**

	Dhaka	Chittagong	Khulna	Rangpur	Sylhet
Gravity model:					
Correlation	0.72	0.69	0.25	0.052	0.14
Radiation model:					
Correlation	0.32	0.73	0.92	0.77	0.86
Time-averaged mobility:					
Correlation	0.97	0.99	1.0	1.0	1.0
Time-averaged mobility versus seeding 1st April 2017:					
Correlation	0.98	0.99	1.0	1.0	1.0

Correlation between the mean initial dates obtained with the daily mobility, and the mean initial dates obtained with the other mobility approximations: the gravity model, the radiation model, and the time-averaged mobility, for the various seeding scenarios. We also provide the correlation between the time-averaged mobility and the daily mobility when seeding on the 1st of April 2017.

In Panigutti et al. (2017), the authors investigated similarity of epidemic outbreak simulations on two alternative mobility networks in France–one based on census data, and the other based on mobile phone mobility data. They calculated a rank correlation between the arrival dates (defined as the first day of an infectious case) in the different locations for different seeding nodes. They found that the epidemic trajectories on the two networks were more similar the higher the degree and strength of the seeding node. We therefore also calculate the degree and strength of the seeding nodes for the different mobility proxies, provided in Table S3. There does not seem to be any connection between neither the strength nor the degree of the node and the correlations in our simulations. Note that all the seeding upazilas are central upazilas, and that such a trend might not be possible to uncover when investigating only five upazilas. It could also be that other network properties could explain the similarities better.

S10 Early spread with gravity and radiation models

We investigate the early spread with the gravity and radiation model approximations, for the different seeding settings, by plotting the prevalence in each upazila 2, 5, 10, 20, 40, and 50 days after the initial seeding date.

S10.1 2017 simulation

The early spatial spread when seeding according to the early cases of 2017 is given in Figure S9 for the gravity model, and in Figure S10 for the radiation model. We clearly see that the epidemic spreads much faster in the gravity model setting than in the mobile phone mobility setting, and more slowly in the radiation model setting.

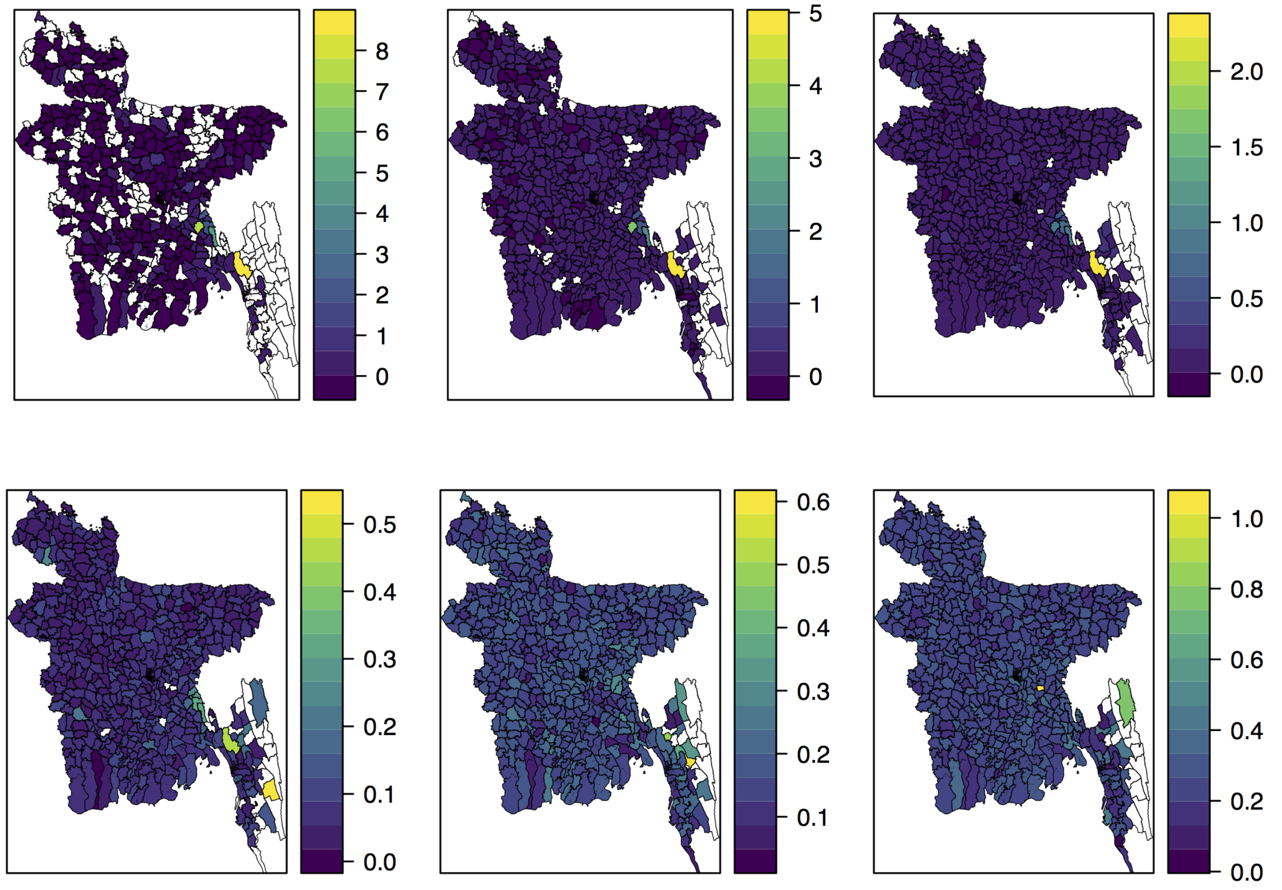

Figure S9: **Gravity model, 2017**. Mean prevalence per 100 000 residents in the different upazilas when seeding according to the 2017 epidemic, a) 2, b) 5, c) 10, d) 20, e) 40, and f) 50 days after the seeding event, using the gravity model to estimate human mobility. Note that the scale is different for all days.

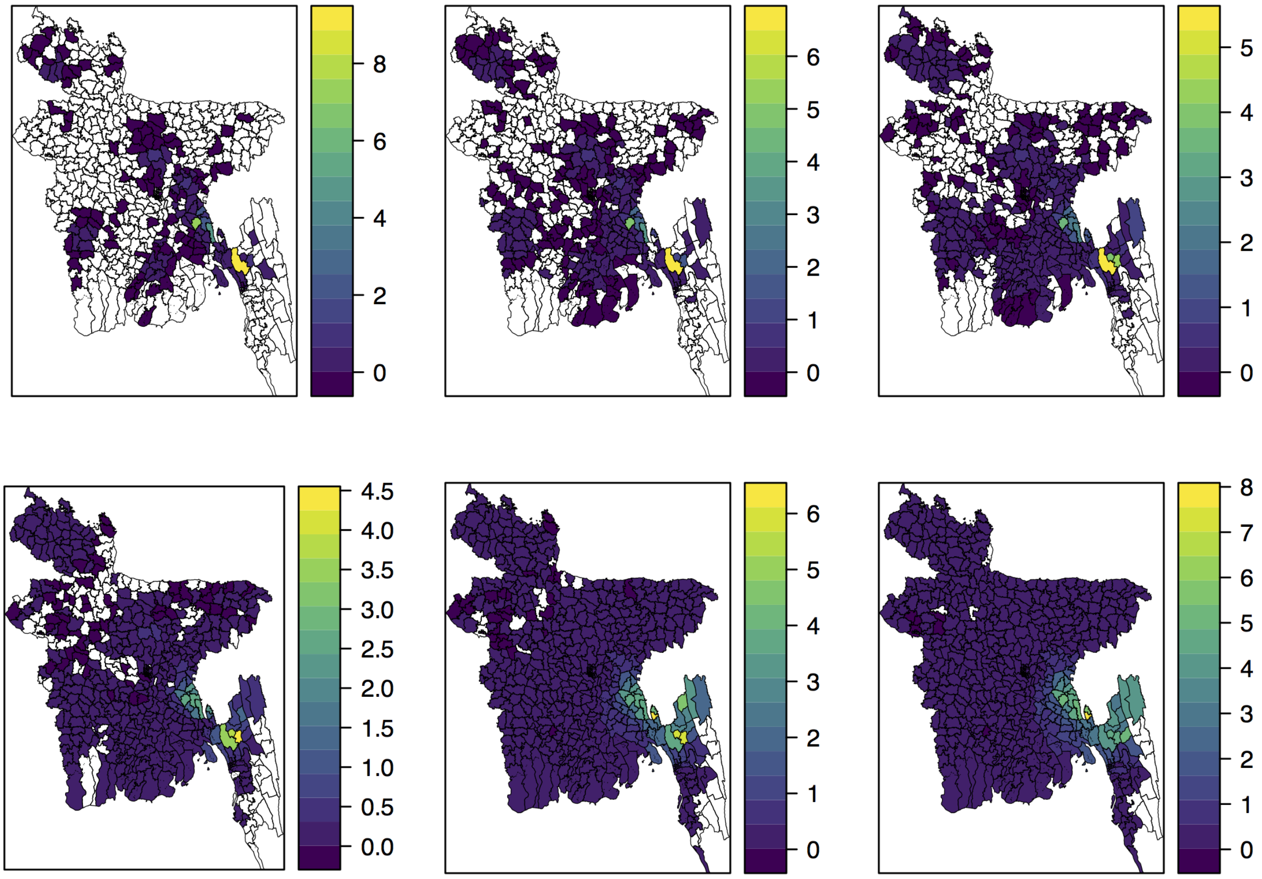

Figure S10: **Radiation model, 2017**. Mean prevalence per 100 000 residents in the different upazilas when seeding according to the 2017 epidemic, a) 2, b) 5, c) 10, d) 20, e) 40, and f) 50 days after the seeding event, using the radiation model to estimate human mobility. Note that the scale is different for all days.

Table S3: **Centrality of seeding nodes.**

	Centrality measure				
	Dhaka	Chittagong	Khulna	Rangpur	Sylhet
Mobile phone data					
Daily mobility:					
Degree	878	725	687	829	934
Strength	470333	257810	321937	616765	800629
Time-averaged mobility					
Degree	1069	1070	1037	1074	1076
Strength	471805	257550	322276	615469	801174
Absolute degree difference	191	345	350	245	142
Relative degree difference	0.22	0.48	0.51	0.30	0.15
Absolute strength difference	1472	260	339	1296	545
Relative strength difference	0.0031	0.0010	0.0011	0.0021	0.00068
Models					
Gravity model:					
Degree	1076	1076	1076	1076	1076
Strength	1053504	208341	306671	544502	665390
Absolute degree difference	198	351	389	247	142
Relative degree difference	0.23	0.48	0.57	0.30	0.15
Absolute strength difference	583171	49469	15266	72263	135239
Relative strength difference	1.2	0.19	0.047	0.12	0.17
Radiation model:					
Degree	883	651	792	916	967
Strength	322581	227582	254690	589097	922483
Absolute degree difference	5	74	105	87	33
Relative degree difference	0.0057	0.10	0.15	0.10	0.035
Absolute strength difference	147752	30228	67247	27668	121854
Relative strength difference	0.31	0.12	0.21	0.045	0.15

Centrality indices for the seeding nodes in the different mobility networks. The centrality indices are for Sutrapur sadar (Dhaka), Double Mooring (Chittagong), Khulna sadar (Khulna), Rangpur sadar (Rangpur), and Sylhet sadar (Sylhet). For the daily mobility, we provide the mean over the 183 days.

S10.2 Dhaka

The early spread when seeding in Dhaka is given in Figure S11 for the gravity model, and in Figure S12 for the radiation model. Also in this setting, the spread based on the gravity model is much faster than the mobile phone mobility setting. The epidemic spreads early to the upazilas within Dhaka division, but also to many other upazilas of the country. In the radiation model setting, the spread is much slower, and most of the early spread is to the upazilas in Dhaka division.

S10.3 Chittagong

The early spread when seeding in Chittagong is given in Figure S13 for the gravity model, and in Figure S14 for the radiation model. Again, the epidemic spreads quickly under the gravity model, both to upazilas of Chittagong, but also to many other upazilas of the country. In the radiation model, the early spread is much more restricted to Chittagong division, and the

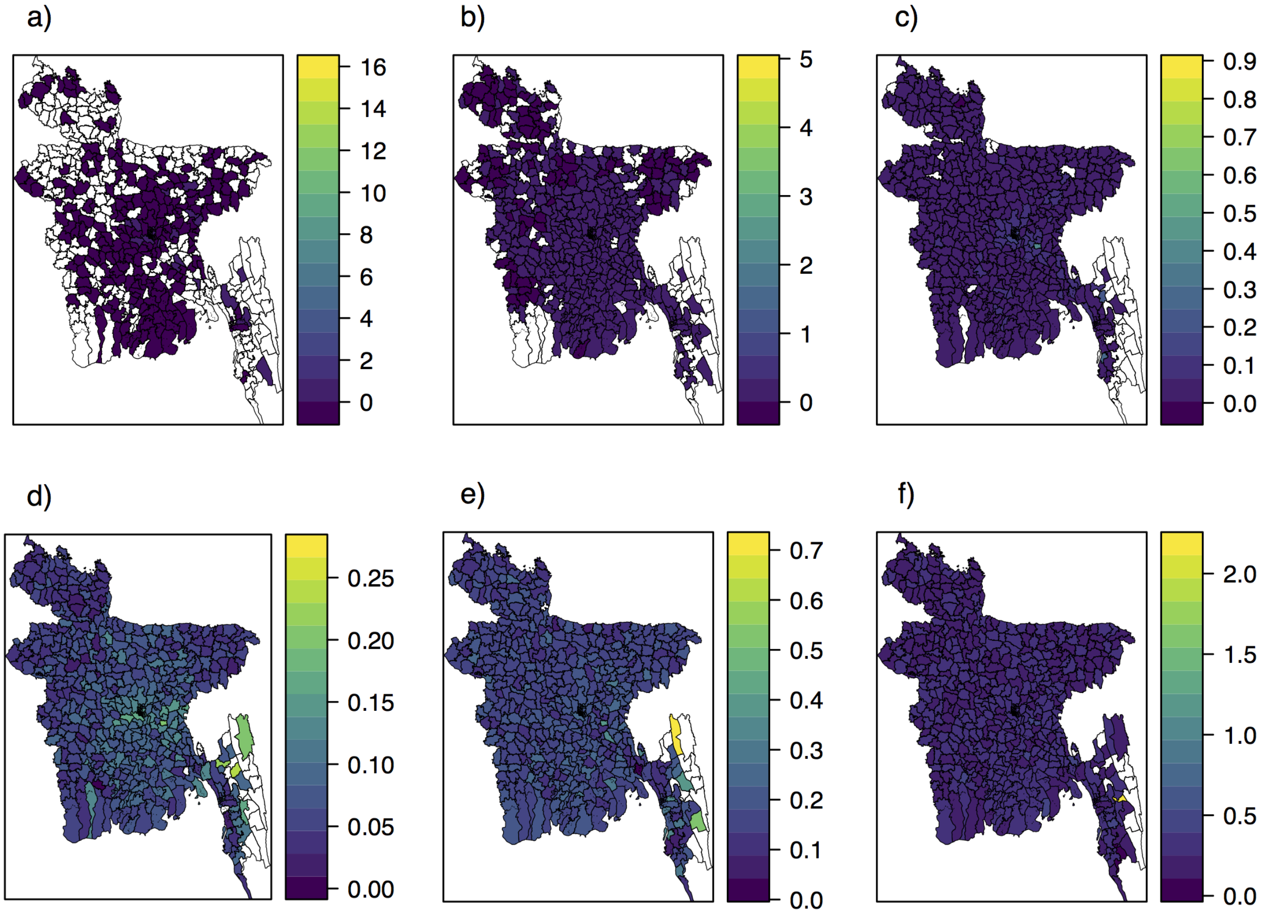

Figure S11: **Gravity model, Dhaka**. Mean prevalence per 100 000 residents in the different upazilas when seeding in Dhaka, a) 2, b) 5, c) 10, d) 20, e) 40, and f) 50 days after the seeding event, using the gravity model to estimate human mobility. Note that the scale is different for all days.

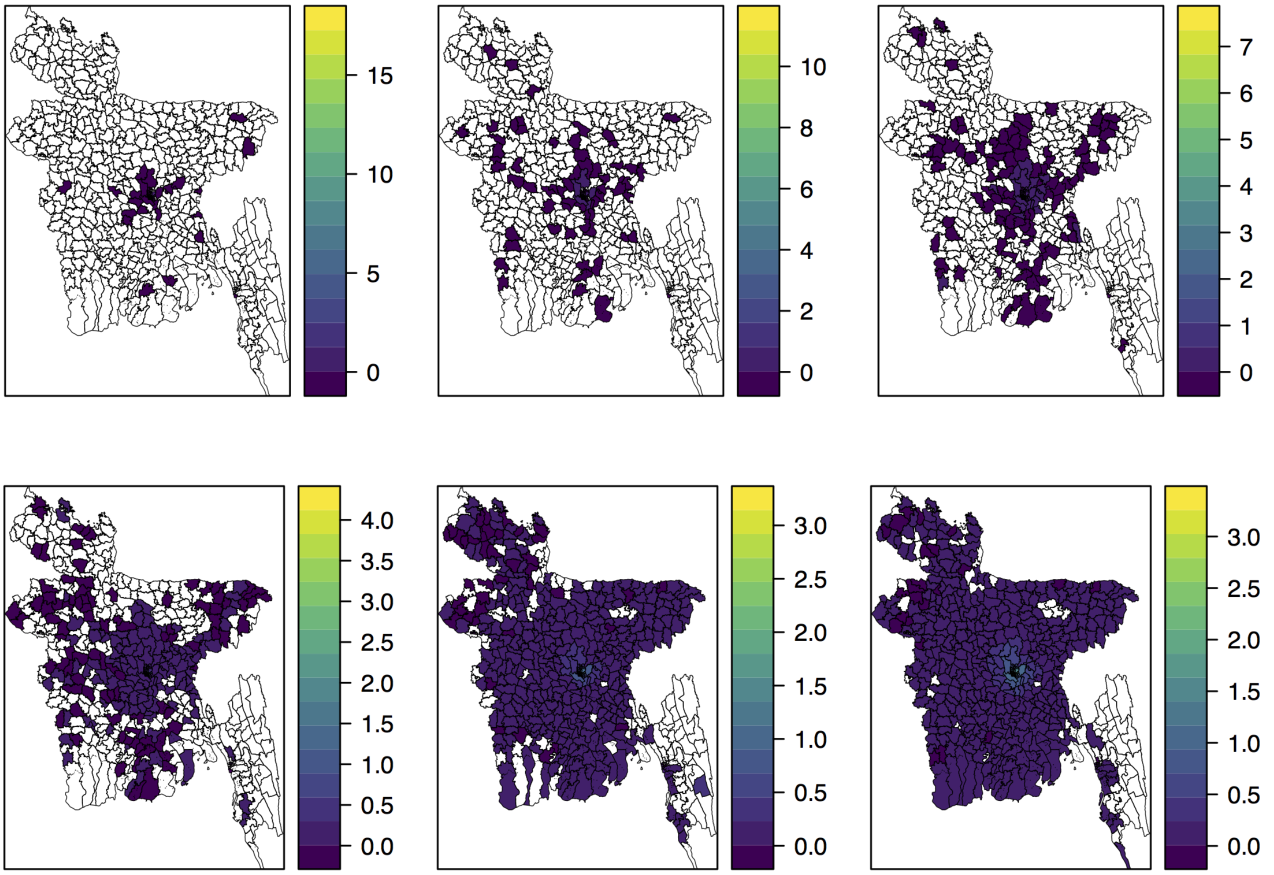

Figure S12: **Radiation model, Dhaka**. Mean prevalence per 100 000 residents in the different upazilas when seeding in Dhaka, a) 2, b) 5, c) 10, d) 20, e) 40, and f) 50 days after the seeding event, using the radiation model to estimate human mobility. Note that the scale is different for all days.

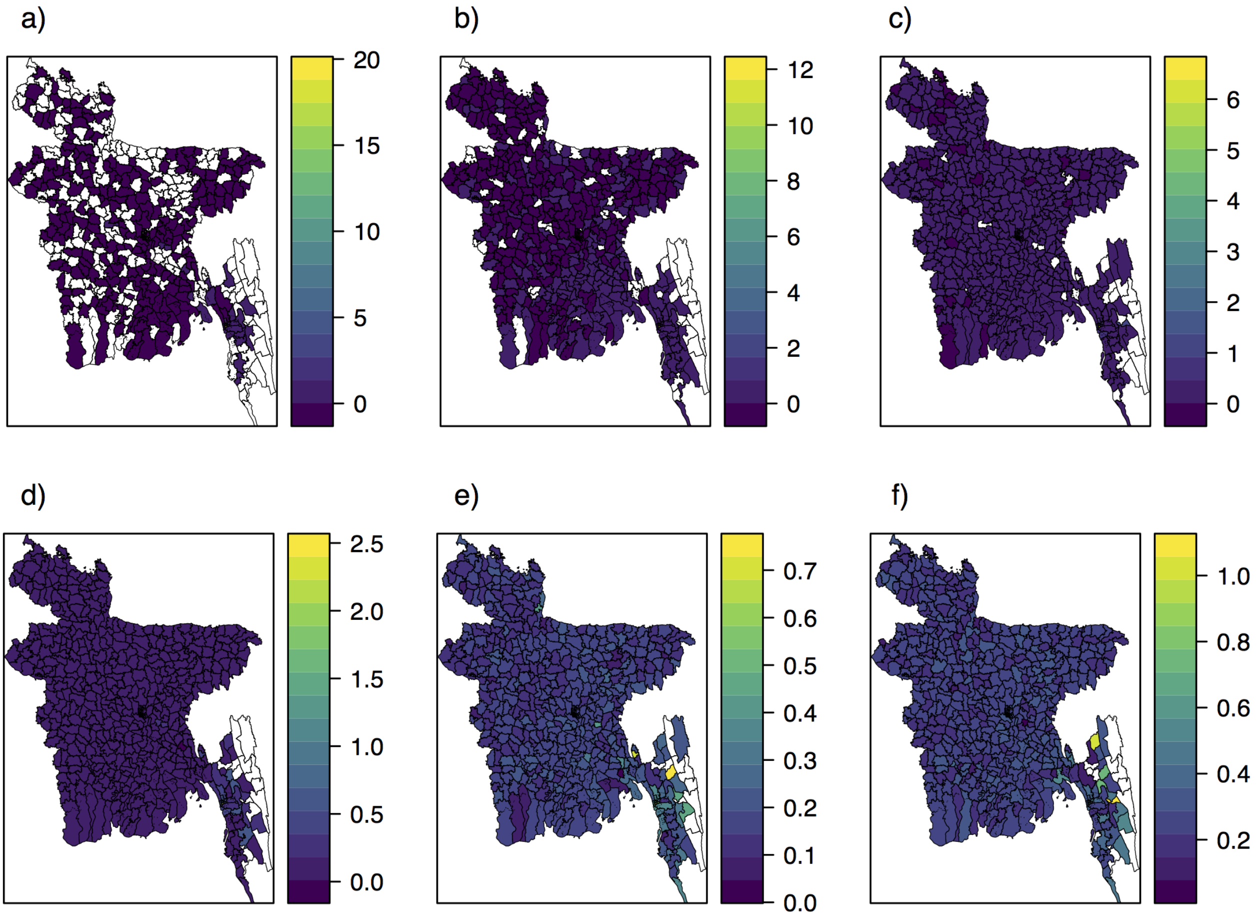

Figure S13: **Gravity model, Chittagong**. Mean prevalence per 100 000 residents in the different upazilas when seeding in Chittagong, a) 2, b) 5, c) 10, d) 20, e) 40, and f) 50 days after the seeding event, using the gravity model to estimate human mobility. Note that the scale is different for all days.

epidemic spreads more slowly than with mobile phone mobility and the gravity model. Dhaka city is also hit early.

S10.4 Khulna

The early spread when seeding in Khulna is given in Figure S15 for the gravity model, and in Figure S16 for the radiation model. Also when seeding in Khulna, the disease spreads quickly under the gravity model, and all the divisions have been reached early. In the radiation model, the spread is again slower than in the mobile phone mobility setting, and the early spread is mainly restricted to Khulna division and upazilas close to the seeding location. The epidemic also spreads quickly to Dhaka city.

S10.5 Rangpur

The early spread when seeding in Rangpur is given in Figure S17 for the gravity model, and in Figure S18 for the radiation model. In the gravity model, the epidemic spreads more quickly than in the mobile phone mobility setting. The early spread is to the upazilas of Rangpur, and Rajshahi, but also to other upazilas around the country. Under the radiation model, the spread is again slower, and the initial spread is radially from the seeding upazila, but also to Dhaka

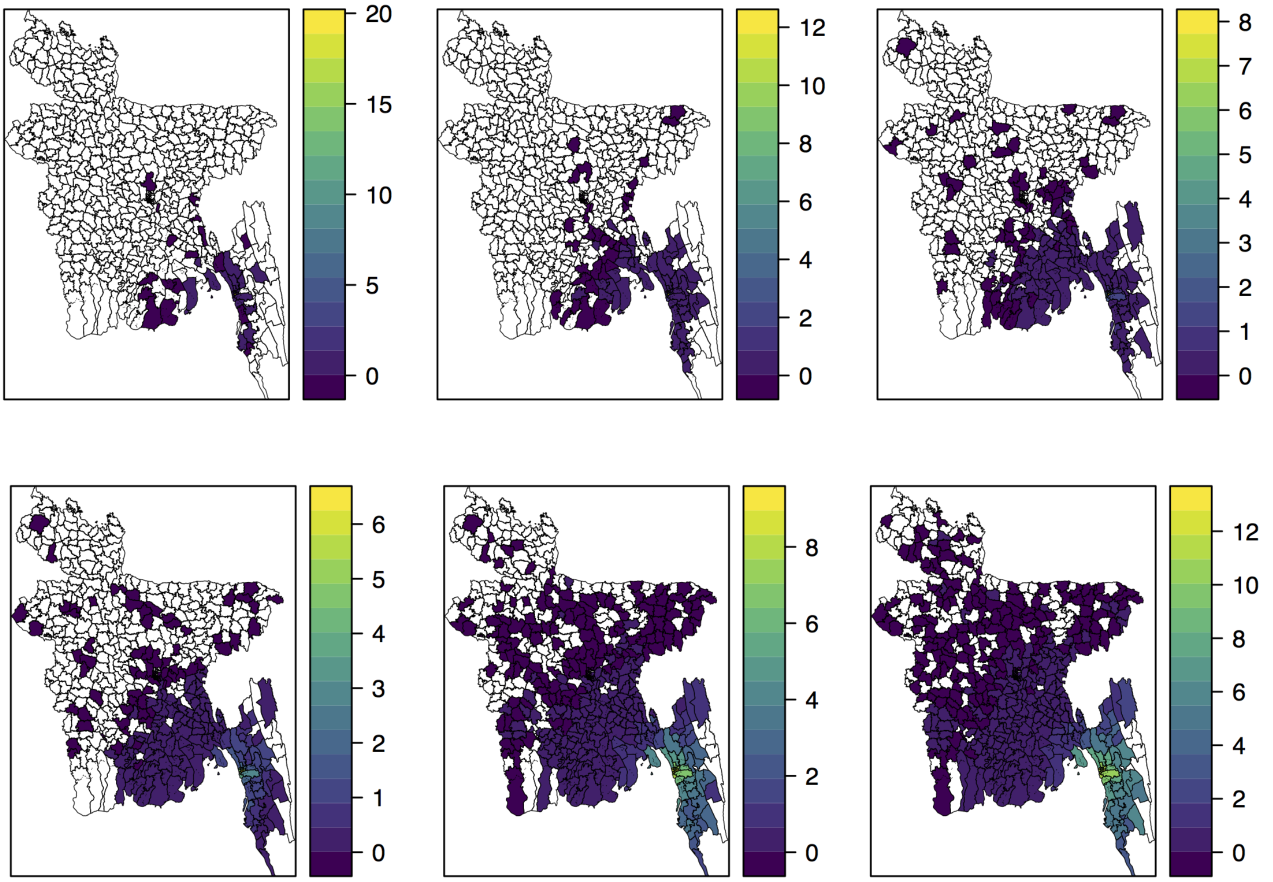

Figure S14: **Radiation model, Chittagong**. Mean prevalence per 100 000 residents in the different upazilas when seeding in Chittagong, a) 2, b) 5, c) 10, d) 20, e) 40, and f) 50 days after the seeding event, using the radiation model to estimate human mobility. Note that the scale is different for all days.

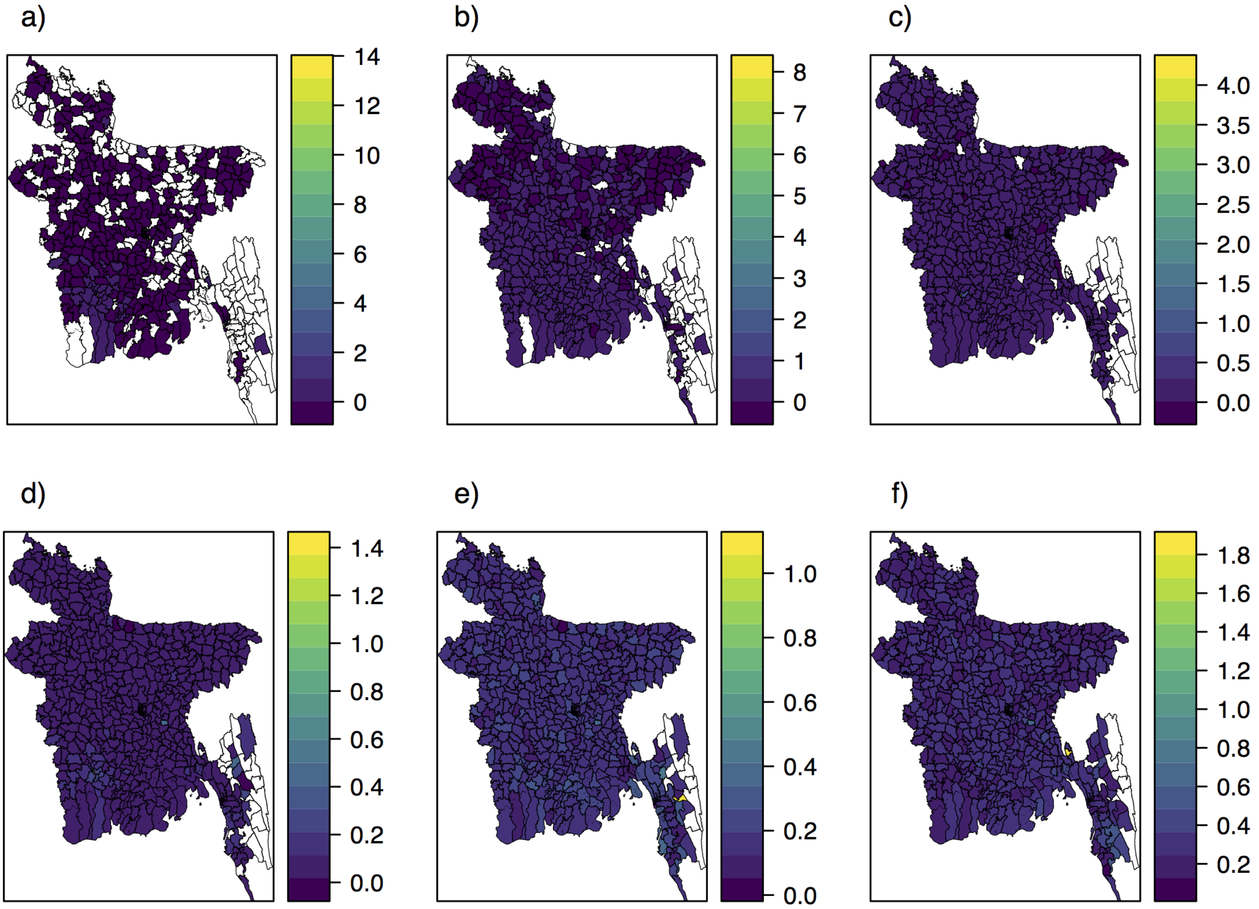

Figure S15: **Gravity model, Khulna**. Mean prevalence per 100 000 residents in the different upazilas when seeding in Khulna, a) 2, b) 5, c) 10, d) 20, e) 40, and f) 50 days after the seeding event, using the gravity model to estimate human mobility. Note that the scale is different for all days.

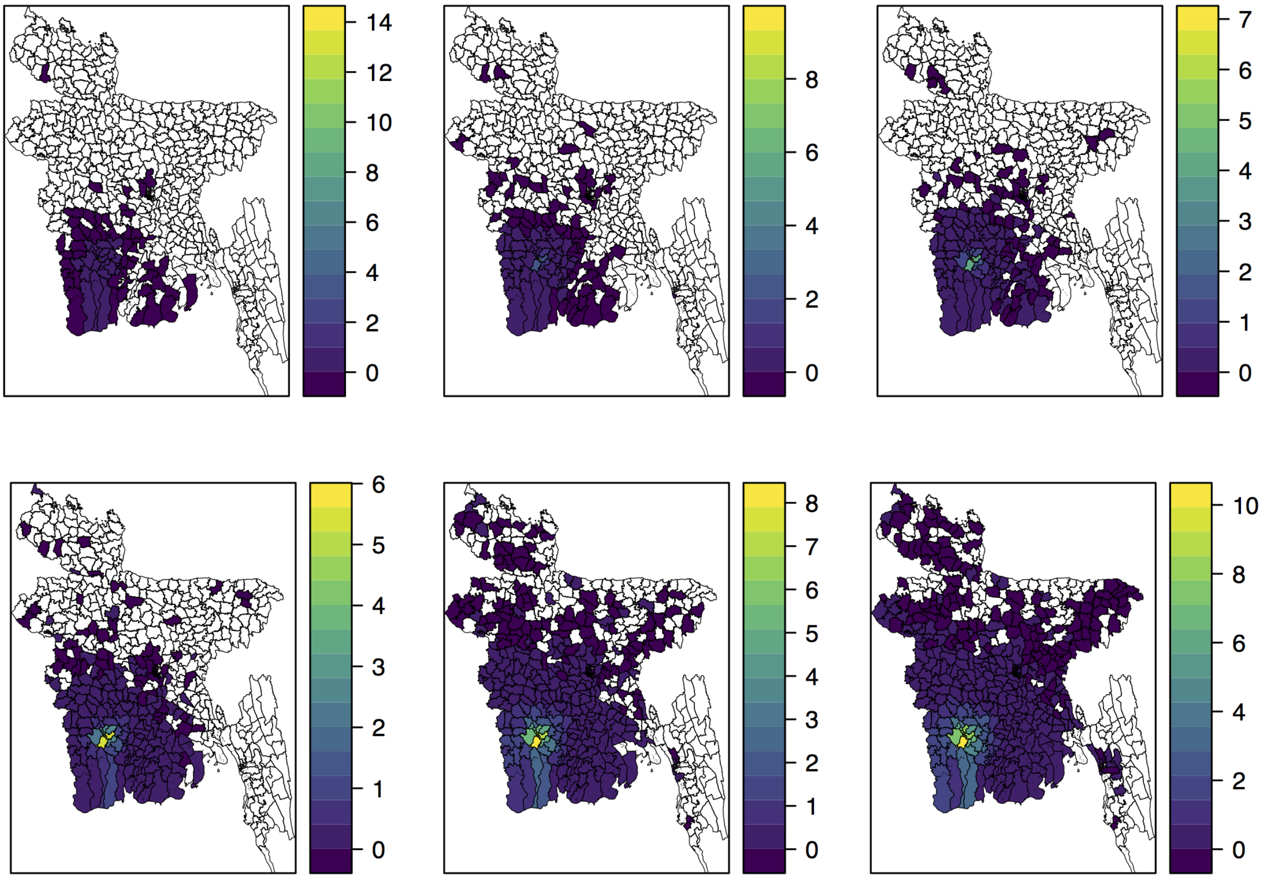

Figure S16: **Radiation model, Khulna**. Mean prevalence per 100 000 residents in the different upazilas when seeding in Khulna, a) 2, b) 5, c) 10, d) 20, e) 40, and f) 50 days after the seeding event, using the radiation model to estimate human mobility. Note that the scale is different for all days.

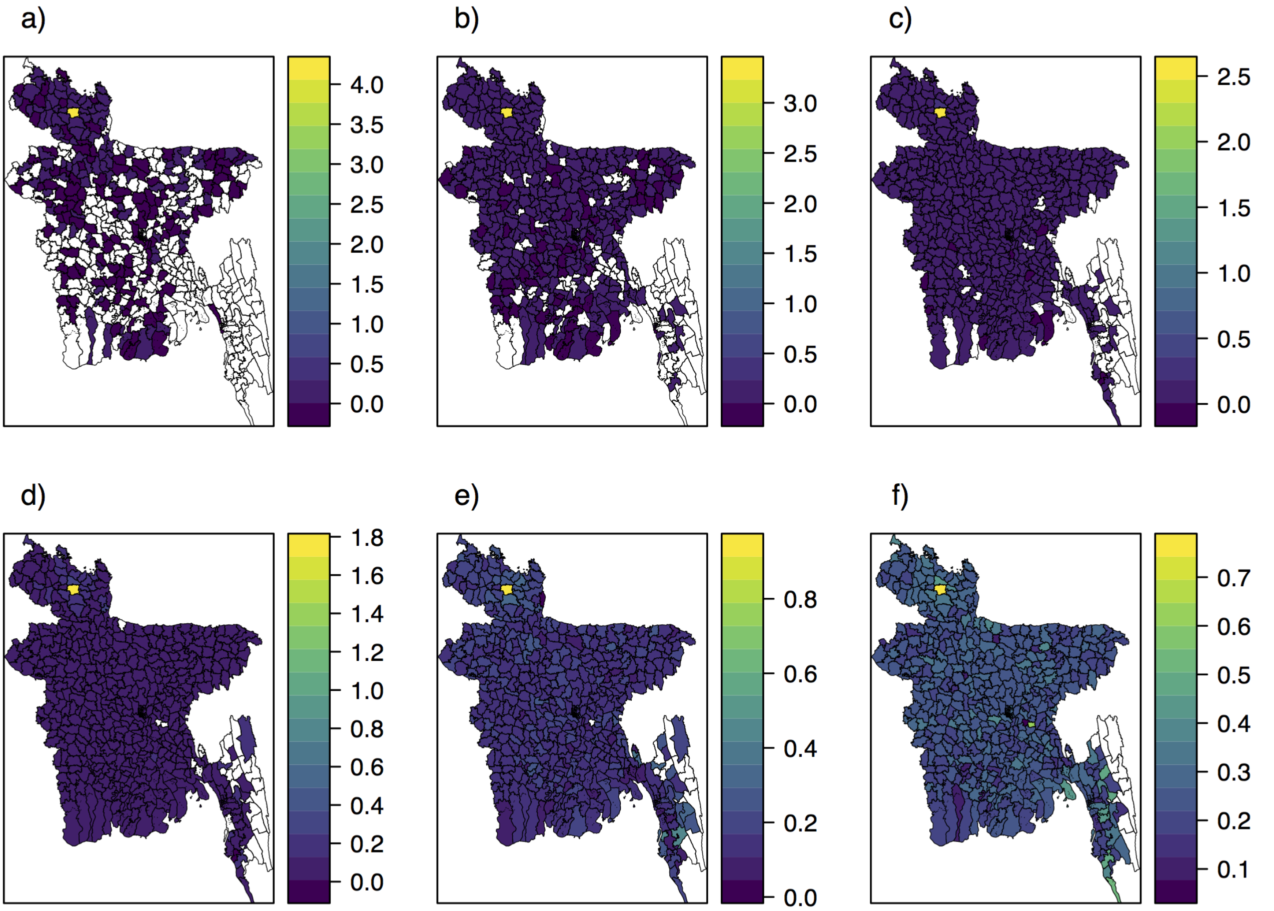

Figure S17: **Gravity model, Rangpur**. Mean prevalence per 100 000 residents in the different upazilas when seeding in Rangpur, a) 2, b) 5, c) 10, d) 20, e) 40, and f) 50 days after the seeding event, using the gravity model to estimate human mobility. Note that the scale is different for all days.

city.

S10.6 Sylhet

The early spatial spread when seeding in Sylhet is given in Figure S19 for the gravity model, and in Figure S20 for the radiation model. Again, the spread using the gravity model is much faster than when using the mobile phone mobility data, and the early spread is both to upazilas of Sylhet, and to other upazilas of the country. The spread is slower in the radiation model setting, and the early spread is to upazilas of Sylhet and to Dhaka city.

S11 Early spread when seeding on April 1st 2017

We investigate how the early spread differs when the daily mobility matrices are used for the first days, that is, when seeding on the 1st of April 2017 instead of the 30th of September 2016. We plot the prevalence in each upazila 2, 5, 10, 20, 40, and 50 days after the initial seeding date for the different seeding scenarios. The plots are given in Figures S21, S22, S23, S24, S25, and S26 when seeding according to the 2017 epidemic, in Dhaka, in Chittagong, in Khulna, in Rangpur, and in Sylhet, respectively. The spreading pattern is very similar to the setting when seeding on

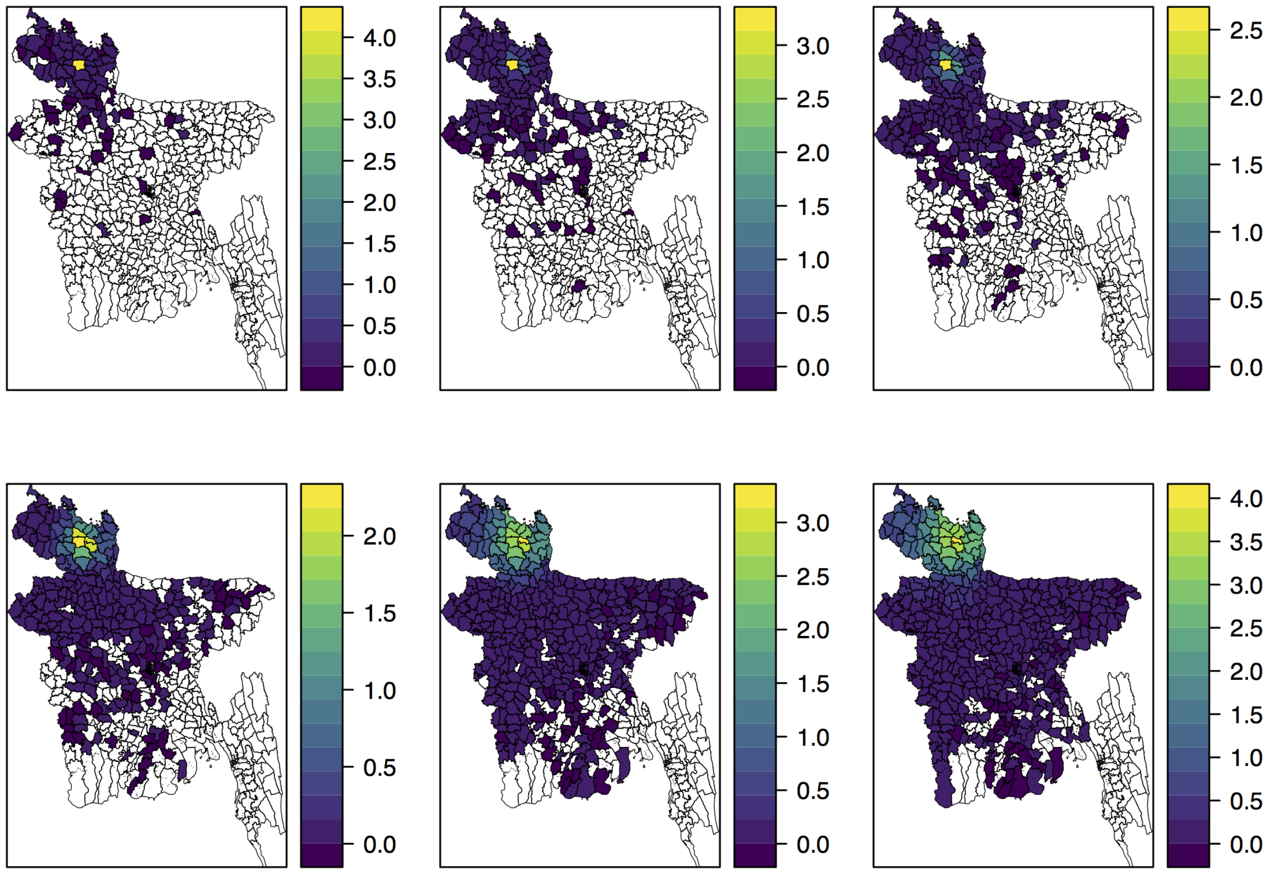

Figure S18: **Radiation model, Rangpur**. Mean prevalence per 100 000 residents in the different upazilas when seeding in Rangpur, a) 2, b) 5, c) 10, d) 20, e) 40, and f) 50 days after the seeding event, using the radiation model to estimate human mobility. Note that the scale is different for all days.

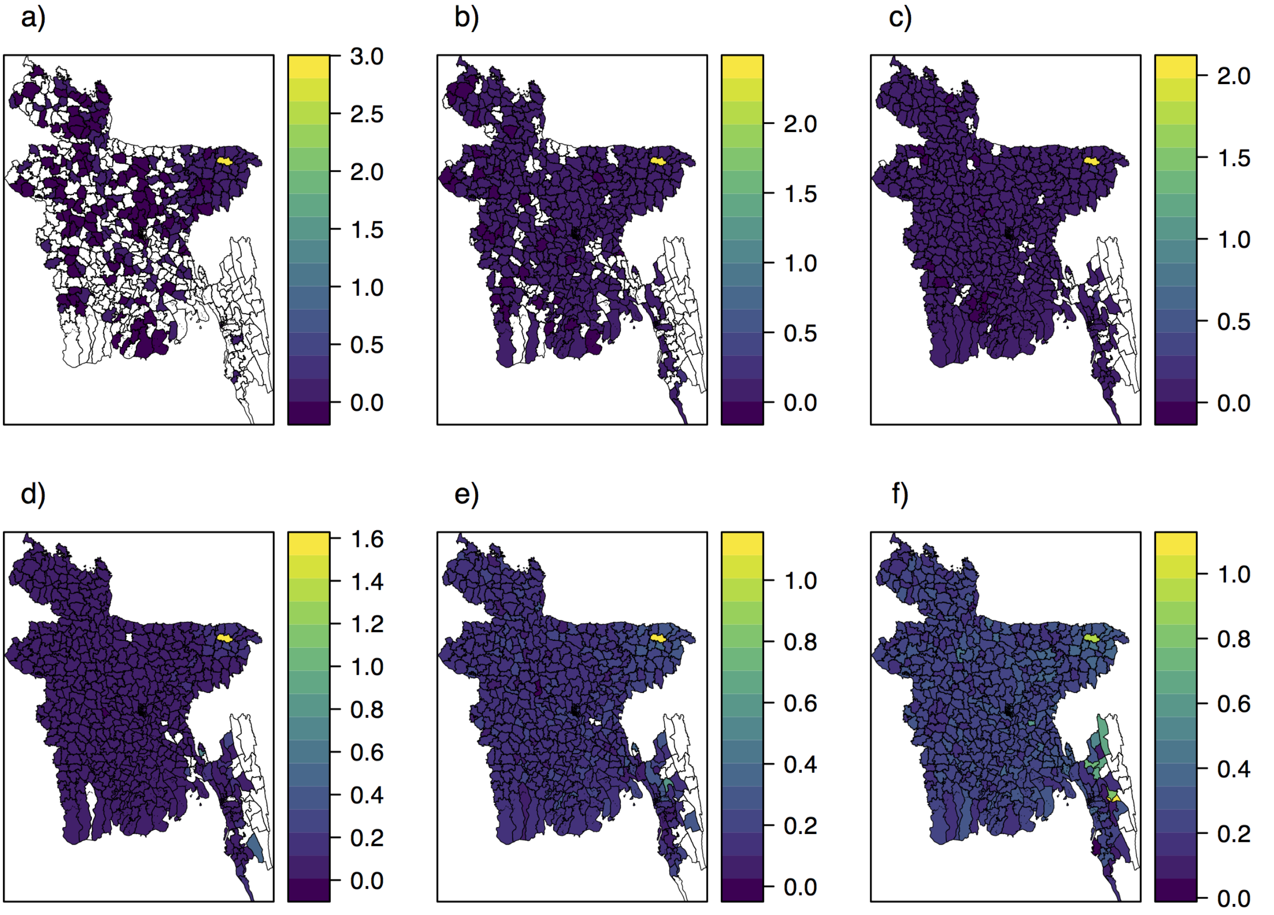

Figure S19: **Gravity model, Sylhet**. Mean prevalence per 100 000 residents in the different upazilas when seeding in Sylhet, a) 2, b) 5, c) 10, d) 20, e) 40, and f) 50 days after the seeding event, using the gravity model to estimate human mobility. Note that the scale is different for all days.

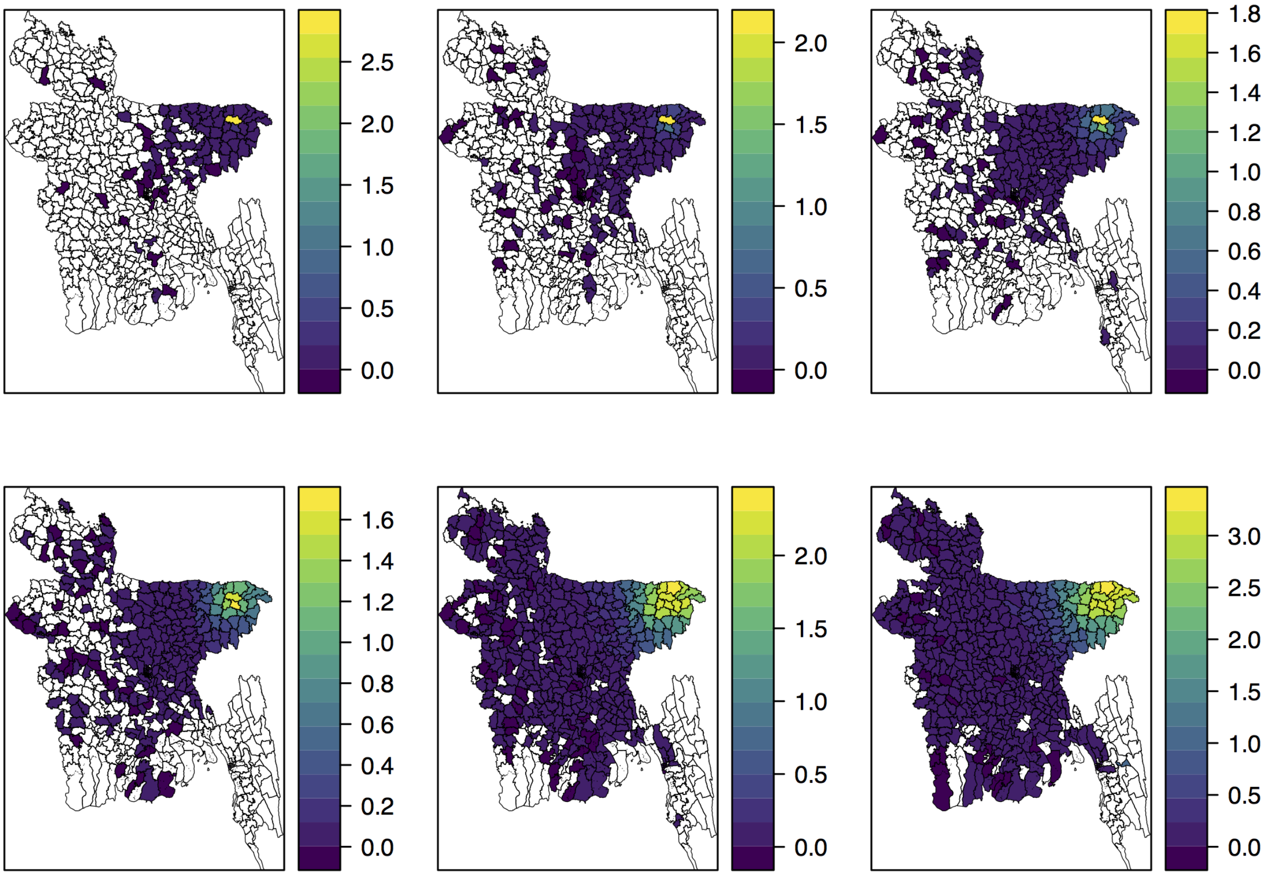

Figure S20: **Radiation model, Sylhet**. Mean prevalence per 100 000 residents in the different upazilas when seeding in Sylhet, a) 2, b) 5, c) 10, d) 20, e) 40, and f) 50 days after the seeding event, using the radiation model to estimate human mobility. Note that the scale is different for all days.

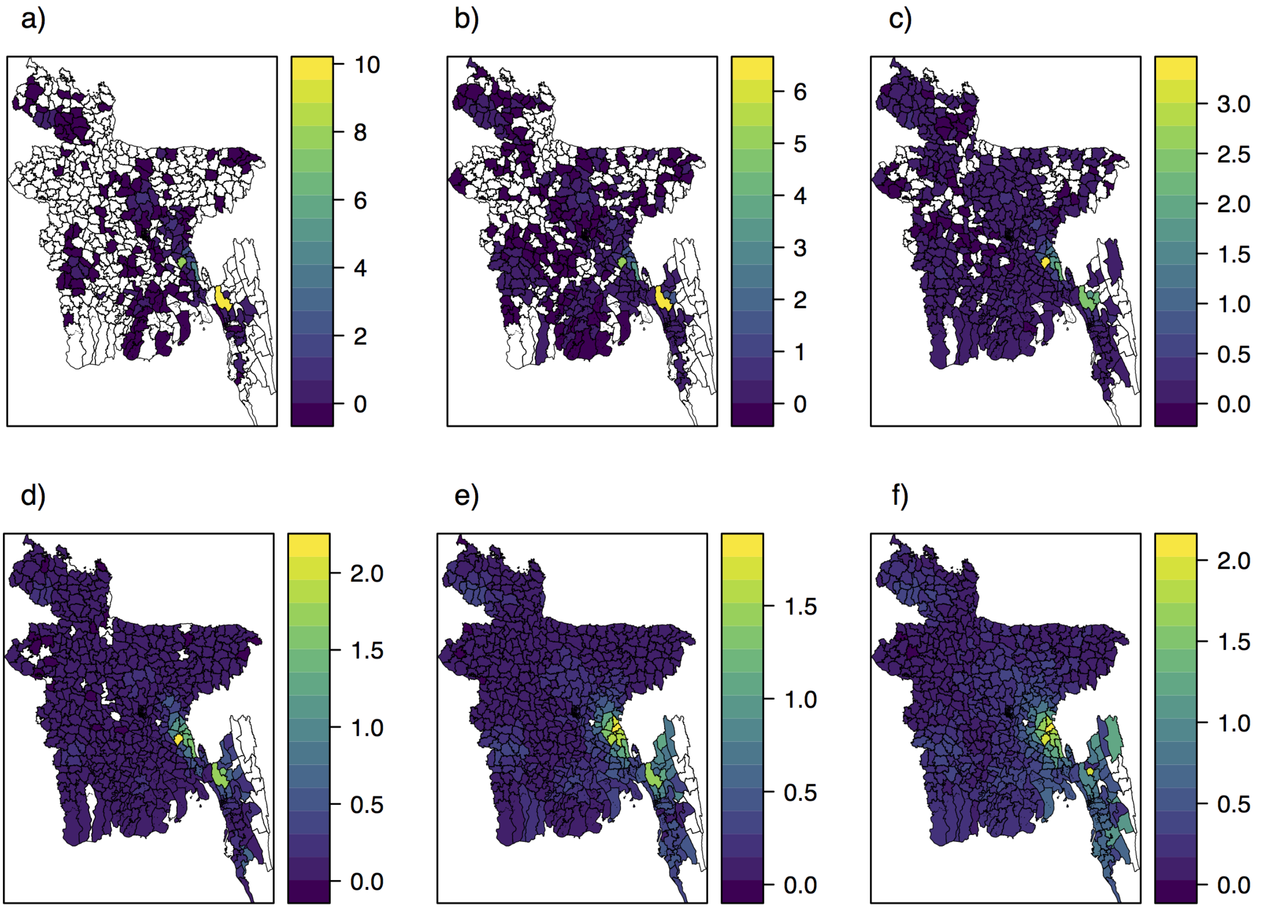

Figure S21: **Daily mobility model seeding 1st of April 2017, 2017**. Mean prevalence per 100 000 residents in the different upazilas when seeding according to the 2017 epidemic on the 1st of April 2017, a) 2, b) 5, c) 10, d) 20, e) 40, and f) 50 days after the seeding event. Note that the scale is different for all days.

the 30th of September, for all the seeding scenarios. Hence the time-averaged mobility is also a good approximation in the initial period of the epidemic.

S12 Initial dates in upazilas

The initial dates in each upazila for the daily mobility setting when seeding on the 30th of September 2016 according to the 2017 epidemic, in Dhaka, in Chittagong, in Khulna, in Rangpur, and in Sylhet are given in Figures S27a-f, respectively. For all seeding scenarios, neighbouring upazilas seem to have similar initial dates. In general, the upazilas in the seeding division have the earliest initial dates. The differences in initial dates between the different upazilas are larger when seeding in Khulna, Rangpur or Sylhet, than when seeding in Dhaka, Chittagong or according to the 2017 epidemic.

We also provide the histogram over the initial dates in the different upazilas in the 100 simulations, provided in Figure S28, by seeding regions. Again we note that the spread is less coherent when seeding in Khulna, Rangpur or Sylhet than when seeding according to the early cases of 2017, in Dhaka or in Chittagong.

The corresponding histograms for the gravity model approximation, the radiation model approximation, the time-averaged mobility and the daily mobility when seeding on the 1st of

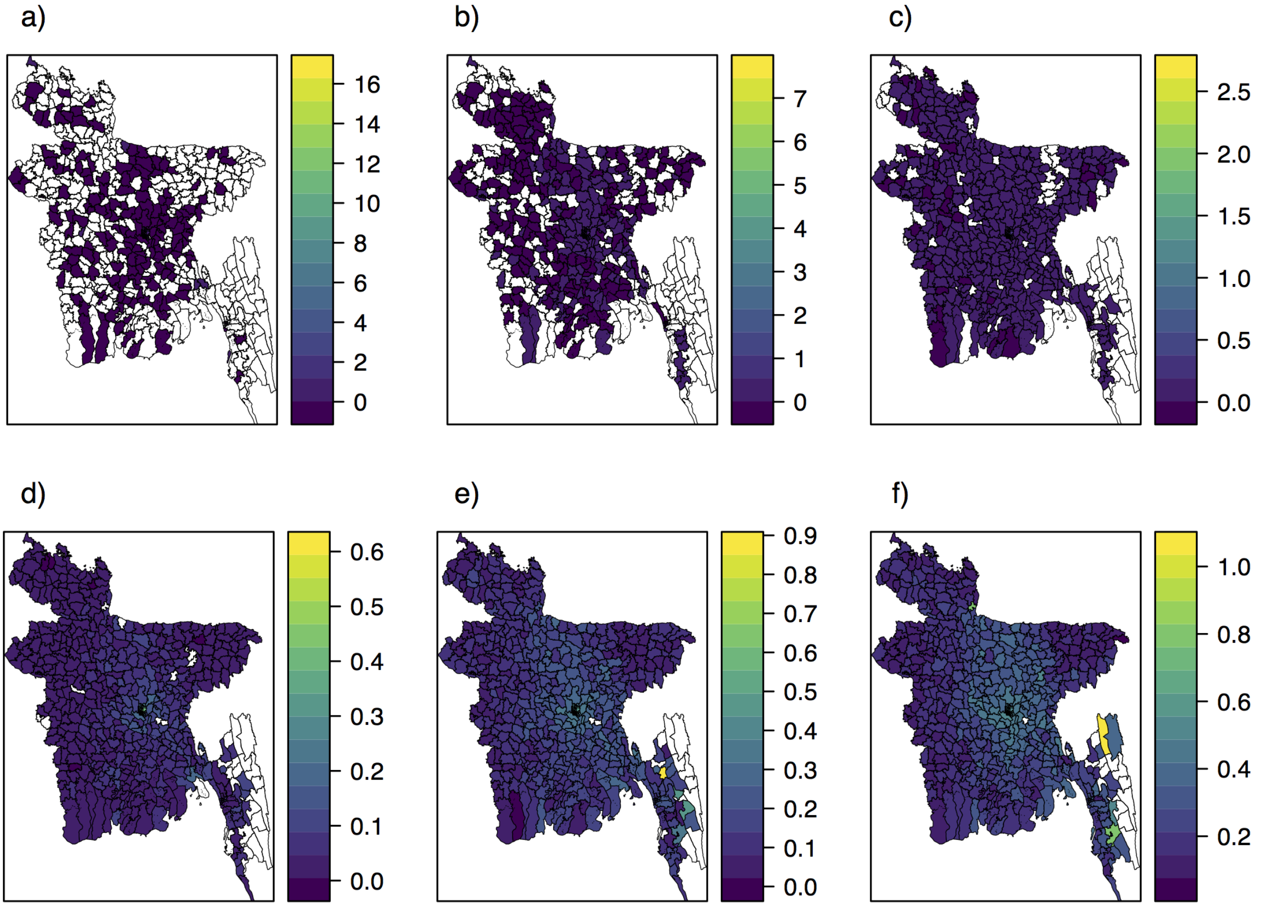

Figure S22: **Daily mobility model seeding 1st of April 2017, Dhaka**. Mean prevalence per 100 000 residents in the different upazilas when seeding in Dhaka on the 1st of April 2017, a) 2, b) 5, c) 10, d) 20, e) 40, and f) 50 days after the seeding event. Note that the scale is different for all days.

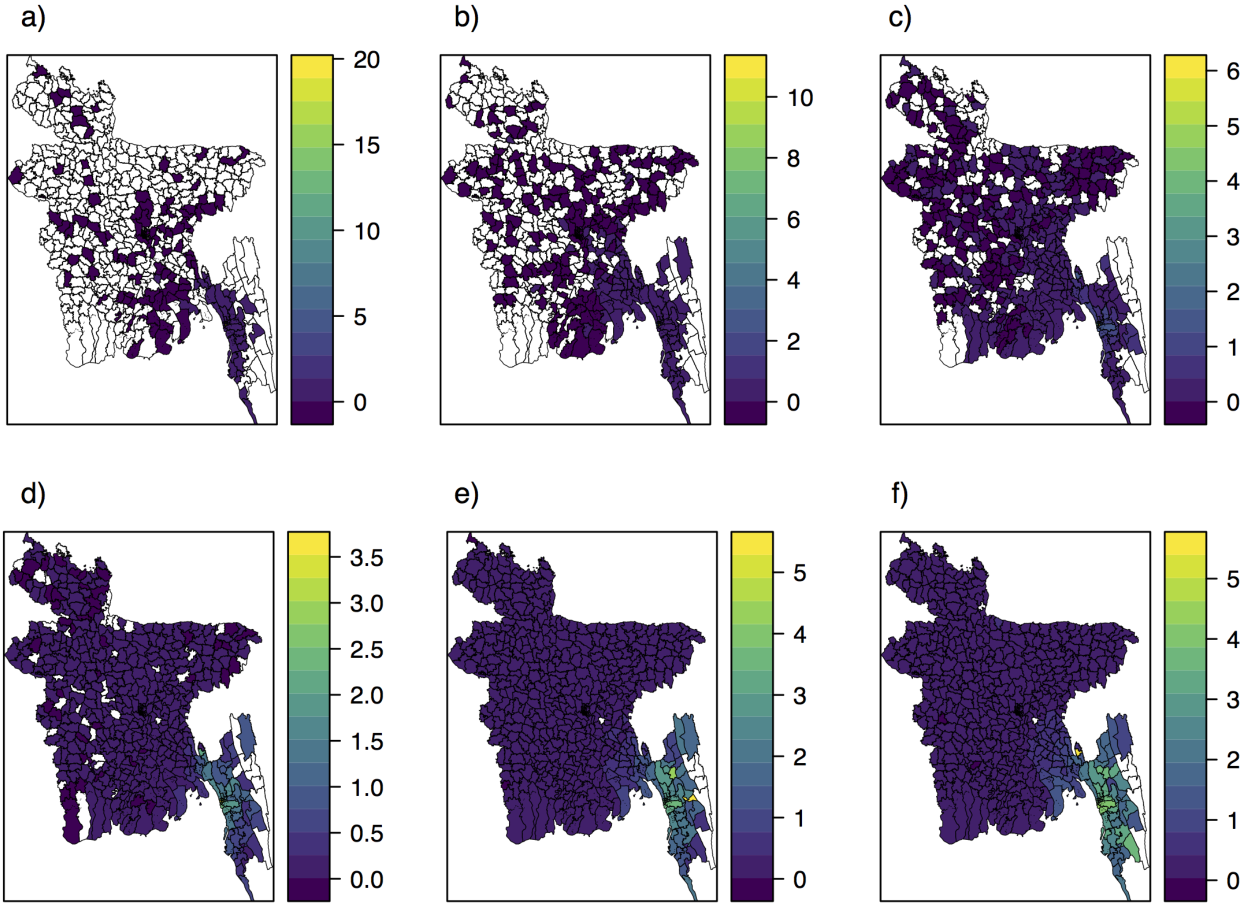

Figure S23: **Daily mobility model seeding 1st of April 2017, Chittagong** Mean prevalence per 100 000 residents in the different upazilas when seeding in Chittagong on the 1st of April 2017, a) 2, b) 5, c) 10, d) 20, e) 40, and f) 50 days after the seeding event. Note that the scale is different for all days.

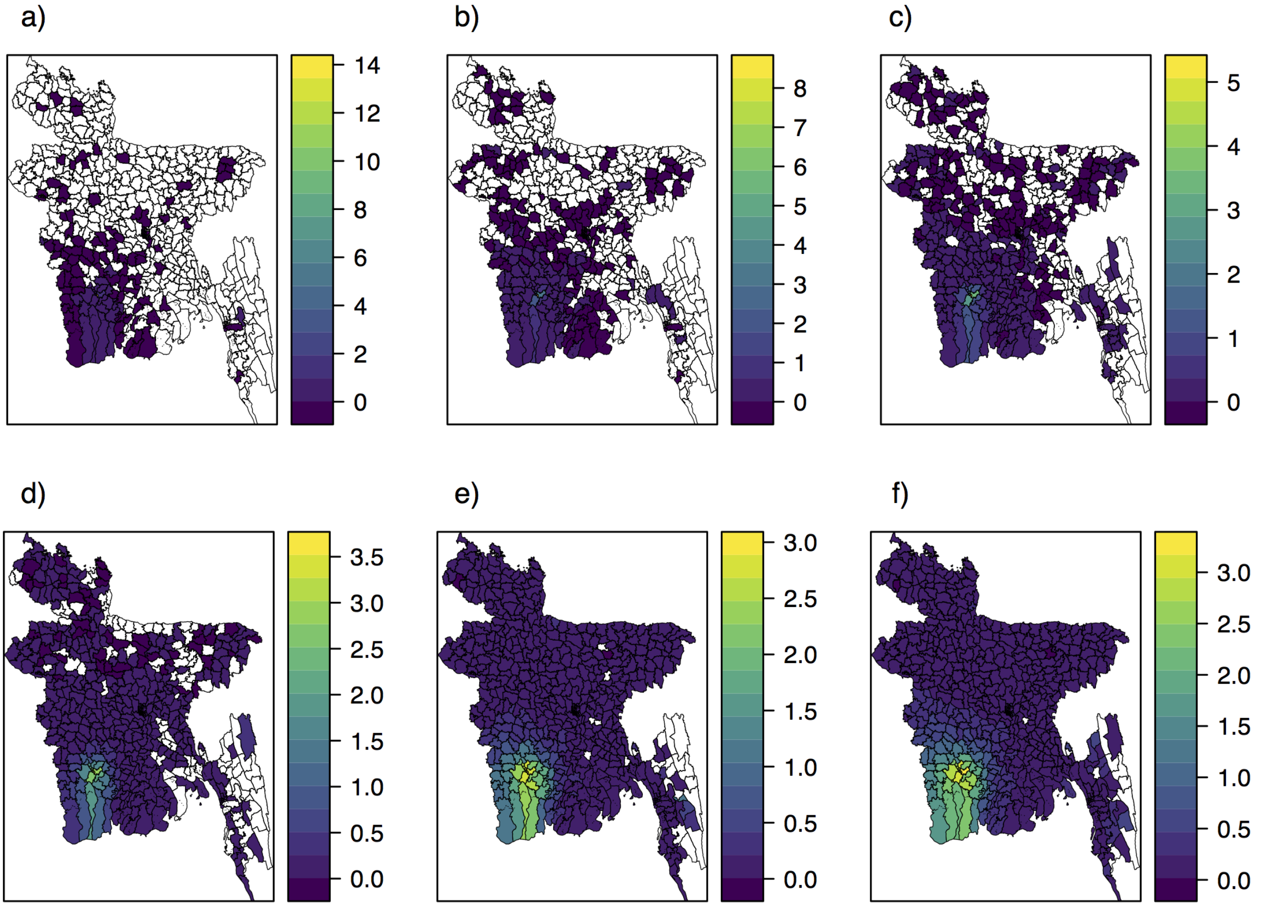

Figure S24: **Daily mobility model seeding 1st of April 2017, Khulna**. Mean prevalence per 100 000 residents in the different upazilas when seeding in Khulna on the 1st of April 2017, a) 2, b) 5, c) 10, d) 20, e) 40, and f) 50 days after the seeding event. Note that the scale is different for all days.

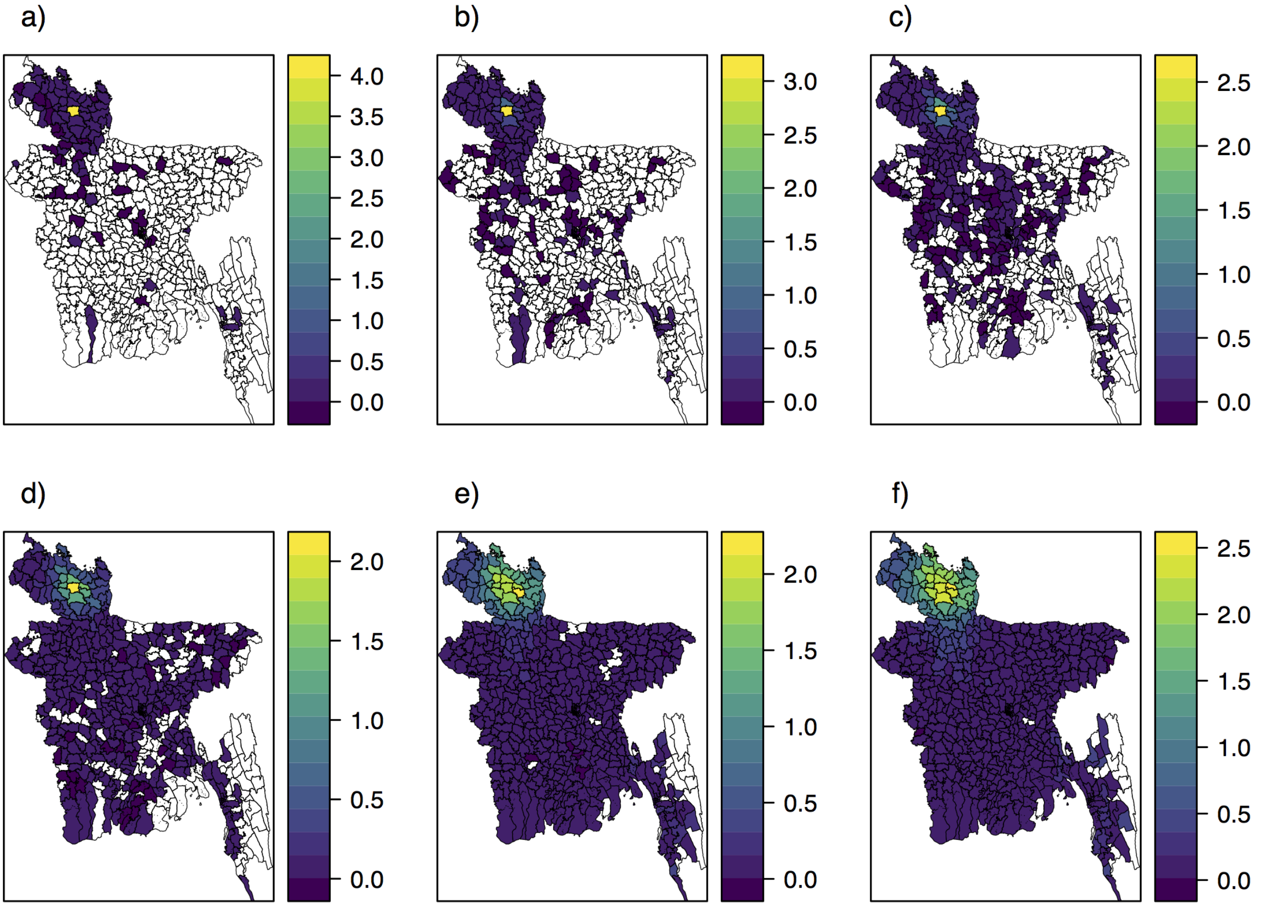

Figure S25: **Daily mobility model seeding 1st of April 2017, Rangpur**. Mean prevalence per 100 000 residents in the different upazilas when seeding in Rangpur on the 1st of April 2017, a) 2, b) 5, c) 10, d) 20, e) 40, and f) 50 days after the seeding event. Note that the scale is different for all days.

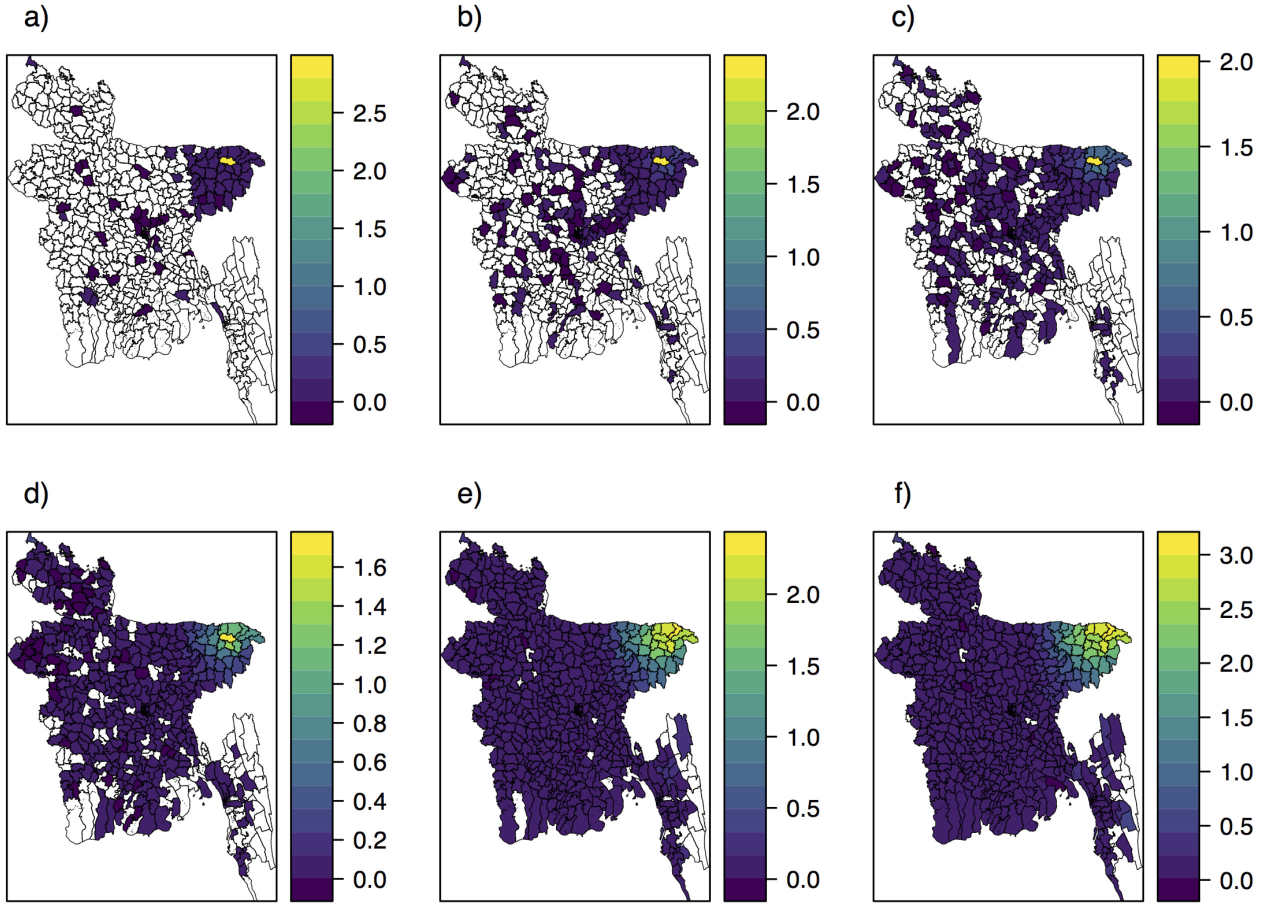

Figure S26: **Daily mobility model seeding 1st of April 2017, Sylhet**. Mean prevalence per 100 000 residents in the different upazilas when seeding in Sylhet on the 1st of April 2017, a) 2, b) 5, c) 10, d) 20, e) 40, and f) 50 days after the seeding event. Note that the scale is different for all days.

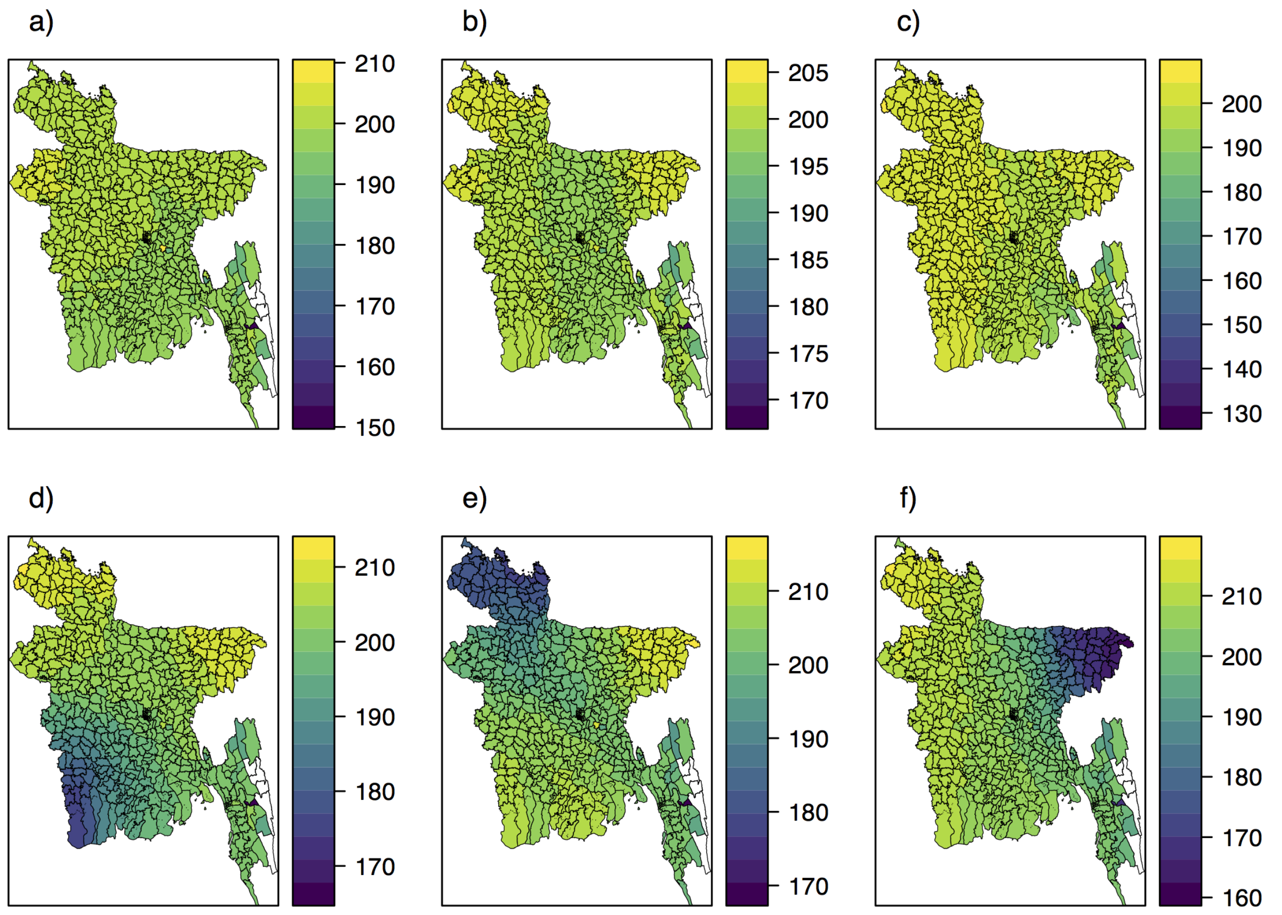

Figure S27: Mean initial dates per upazila for various seeding locations. a) 2017 simulation, b) Dhaka, c) Chittagong, d) Khulna, e) Rangpur f) Sylhet. Note that the scales on the colour bars depend on the seeding location.

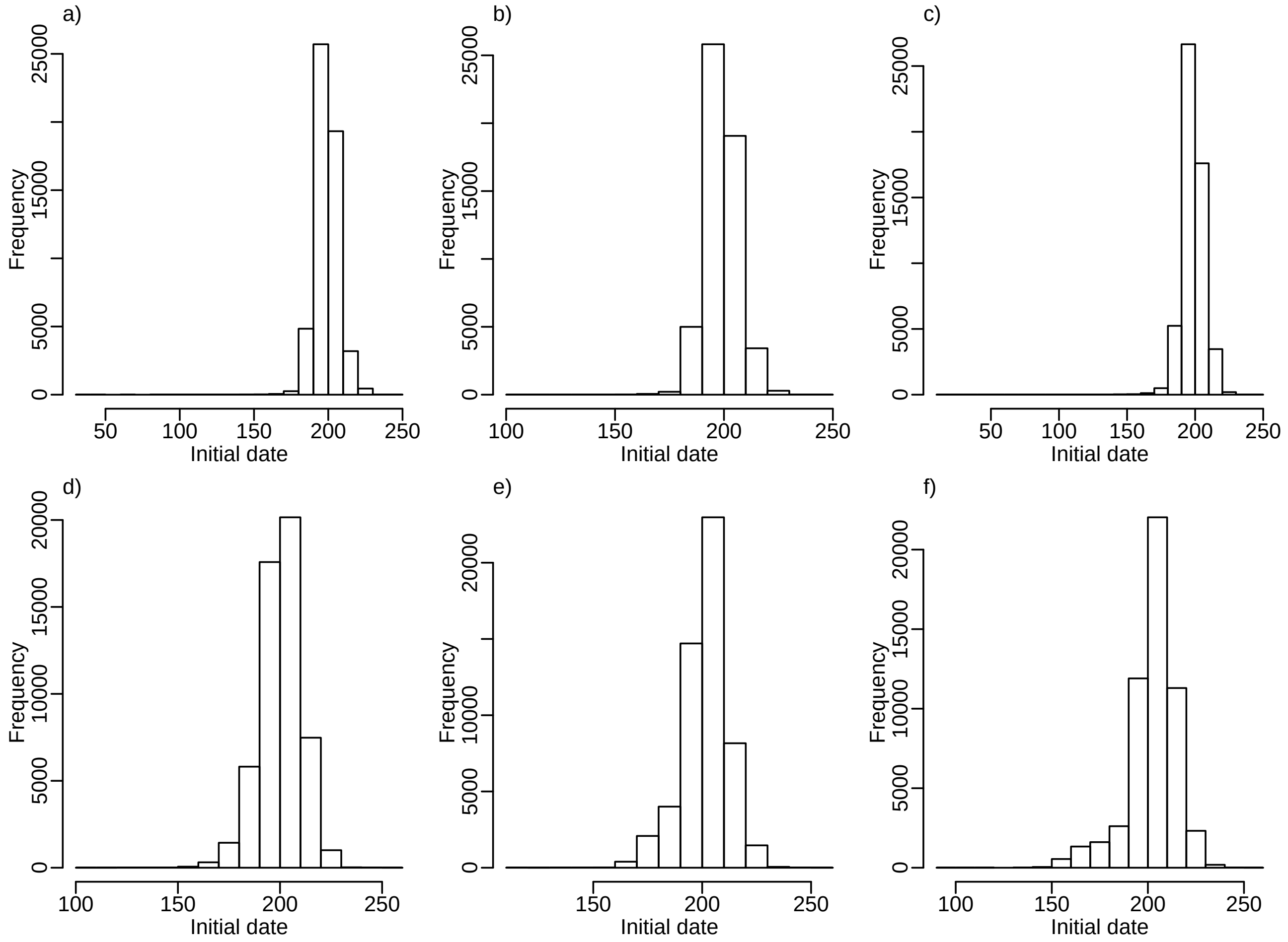

Figure S28: **Daily mobility**: Initial dates distribution over the different upazilas and 100 simulations for various seeding locations. a) 2017 simulation, b) Dhaka, c) Chittagong, d) Khulna, e) Rangpur f) Sylhet. Note that the scales on the y-axes depend on the seeding location.

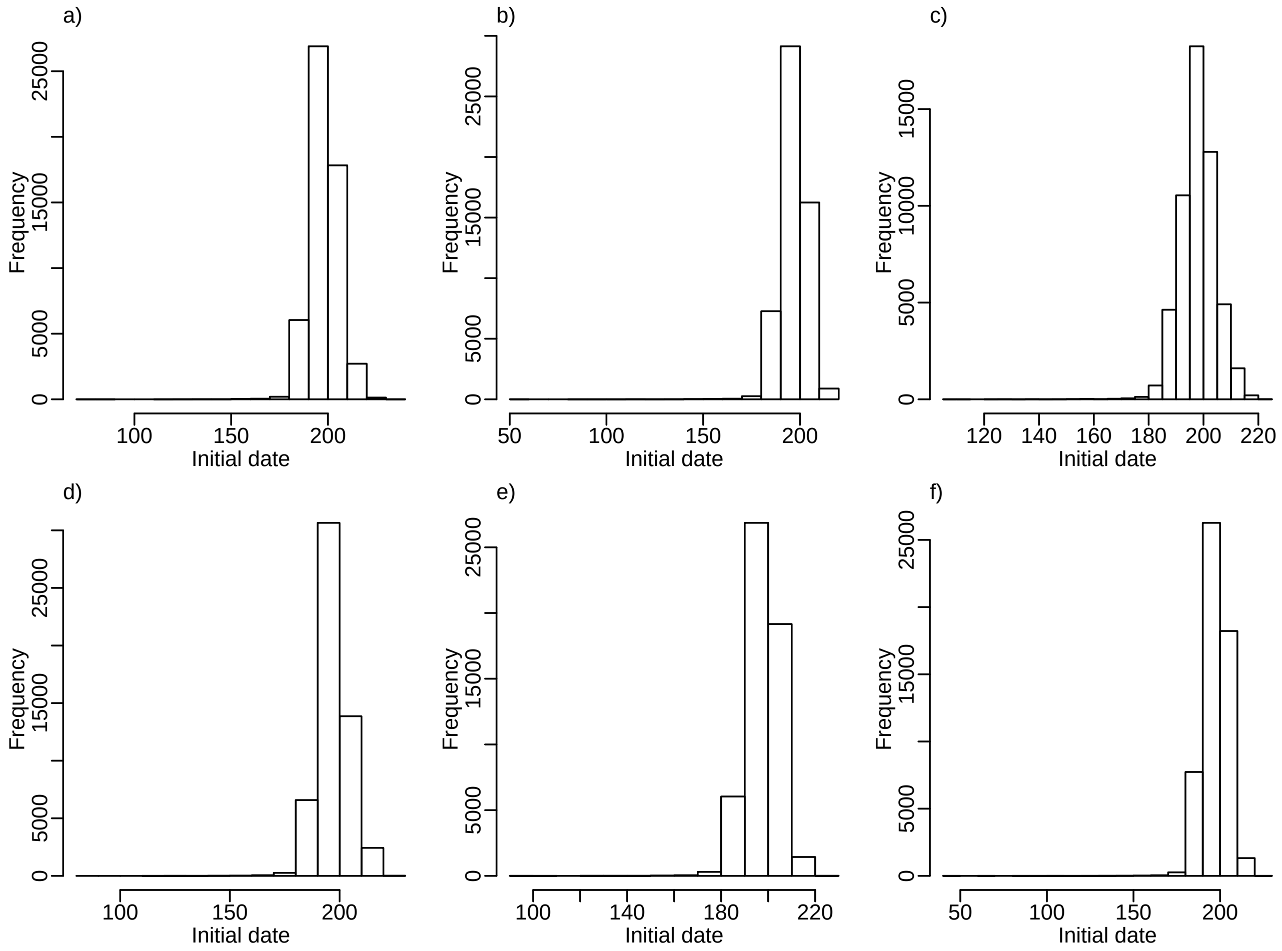

Figure S29: **Gravity model**: Initial dates distribution over the different upazilas and 100 simulations for various seeding locations. a) 2017 simulation, b) Dhaka, c) Chittagong, d) Khulna, e) Rangpur f) Sylhet. Note that the scales on the y-axes depend on the seeding location.

April are given in Figures S29-S32, respectively. The initial dates per upazila under the gravity model are very similar for the different seeding scenarios, while for the radiation model the distributions are much wider than for the daily mobility and the gravity model, and depend more on the seeding locations.

S13 Seeding in Barisal and Rajshahi

For completeness, we also include simulations when seeding in the two remaining divisions, Barisal and Rajshahi, seeding in the upazilas Barisal Sadar and Matihar, respectively. The resulting initial dates in each upazila are given in Figure S33. The disease clearly sparks first in the seeding divisions in both settings. When seeding in Barisal, the disease spreads to Khulna, Chittagong, and Dhaka before spreading to the rest of the country. When seeding in Rajshahi, the disease spreads to Rangpur, Khulna, Chittagong, and Dhaka before spreading to the rest of the country, and the upazilas of Sylhet have the latest initial dates. The early spread when seeding in Barisal is given in Figure S34, and the early spread when seeding in Rajshahi is given in Figure S35. Similar to the spreading pattern for the other seeding locations, the early spread is to upazilas within the seeding divisions, but also to larger cities and in particular to Dhaka city. The distributions of initial dates for each division when seeding in Barisal and Rajshahi are given in Figure S36. In both cases, the seeding division clearly has earlier initial dates

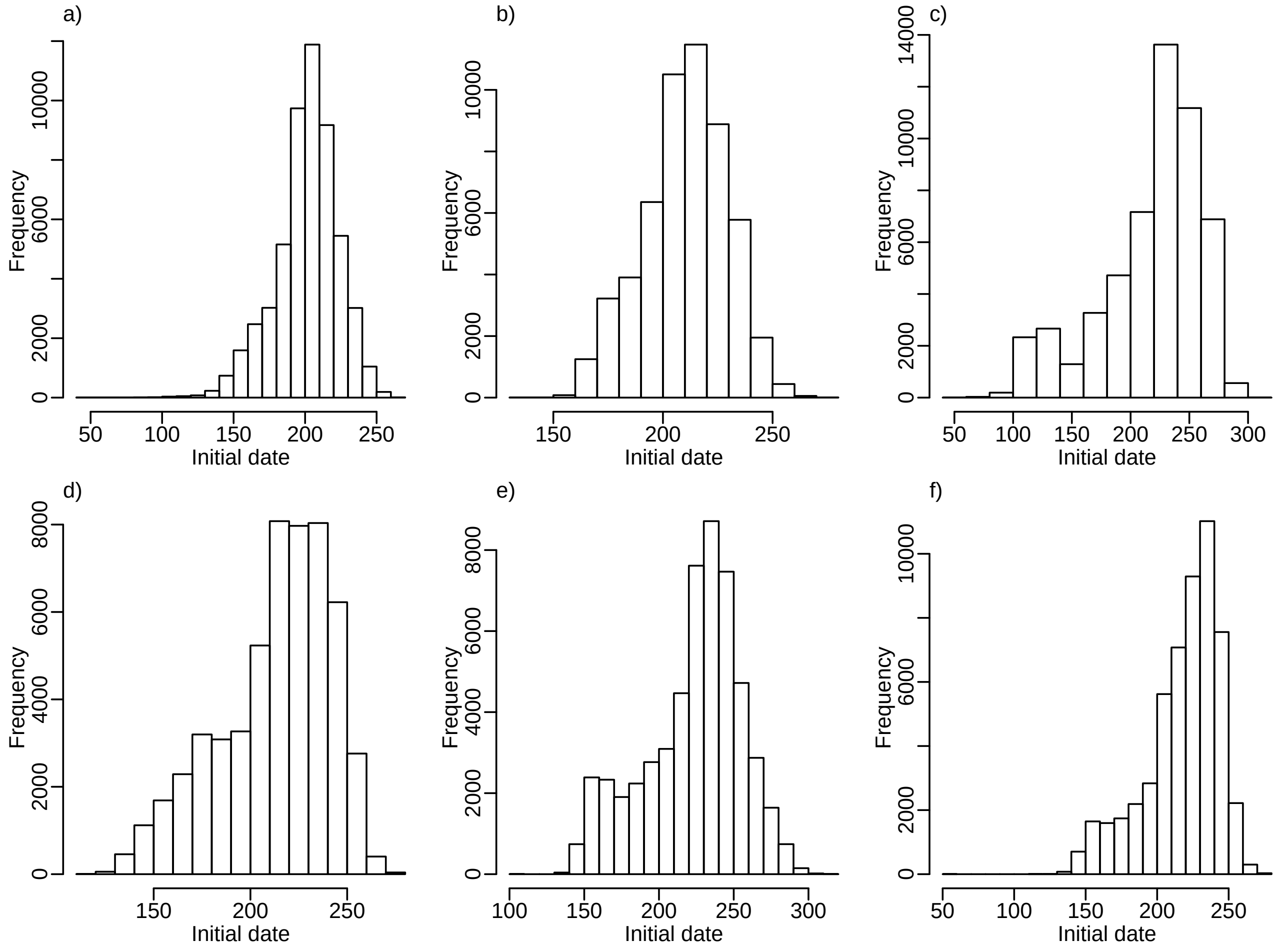

Figure S30: **Radiation model**: Initial dates distribution over the different upazilas and 100 simulations for various seeding locations. a) 2017 simulation, b) Dhaka, c) Chittagong, d) Khulna, e) Rangpur f) Sylhet. Note that the scales on the y-axes depend on the seeding location.

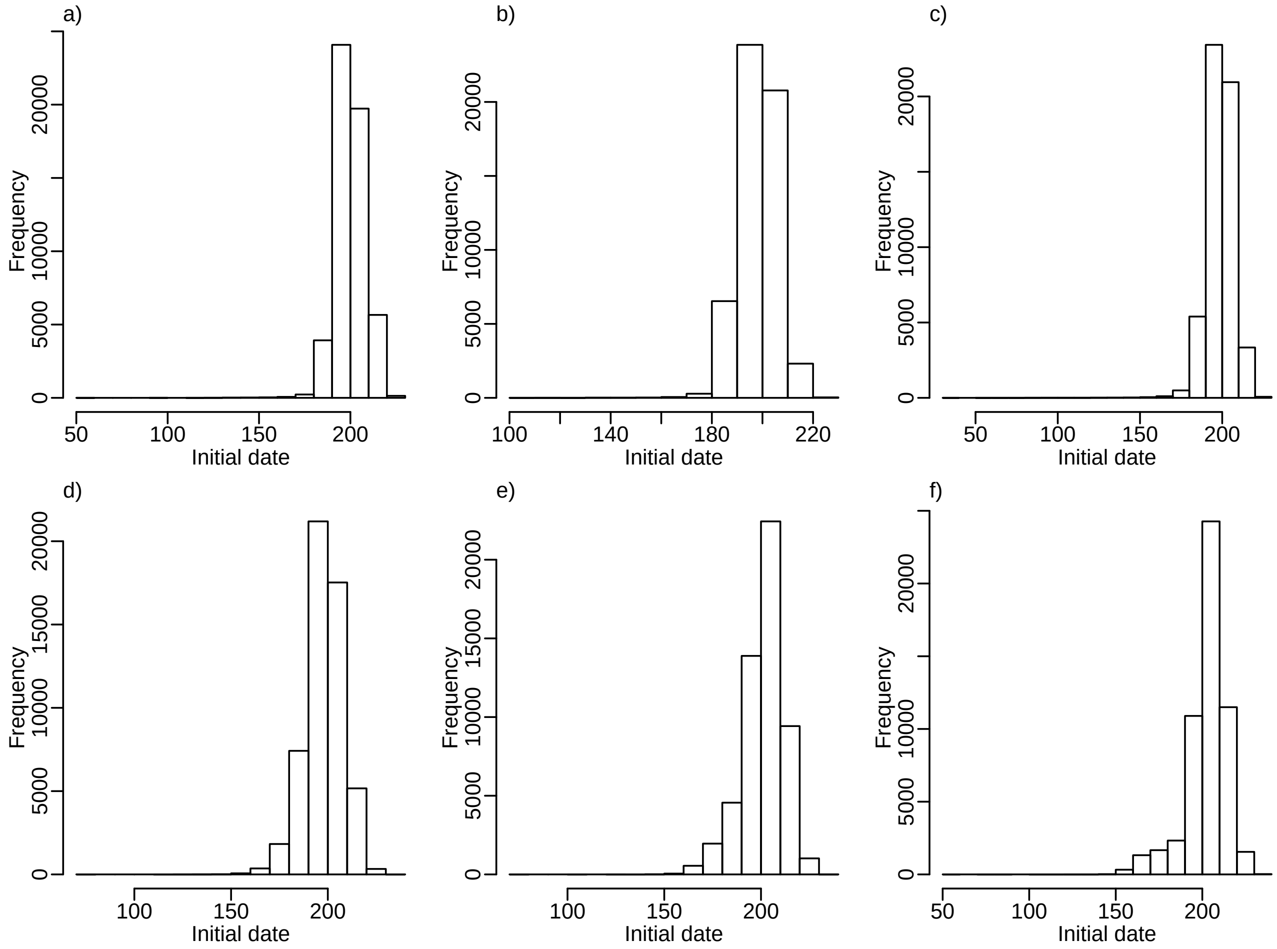

Figure S31: **Time-averaged mobility**: Initial dates distribution over the different upazilas and 100 simulations for various seeding locations. a) 2017 simulation, b) Dhaka, c) Chittagong, d) Khulna, e) Rangpur f) Sylhet. Note that the scales on the y-axes depend on the seeding location.

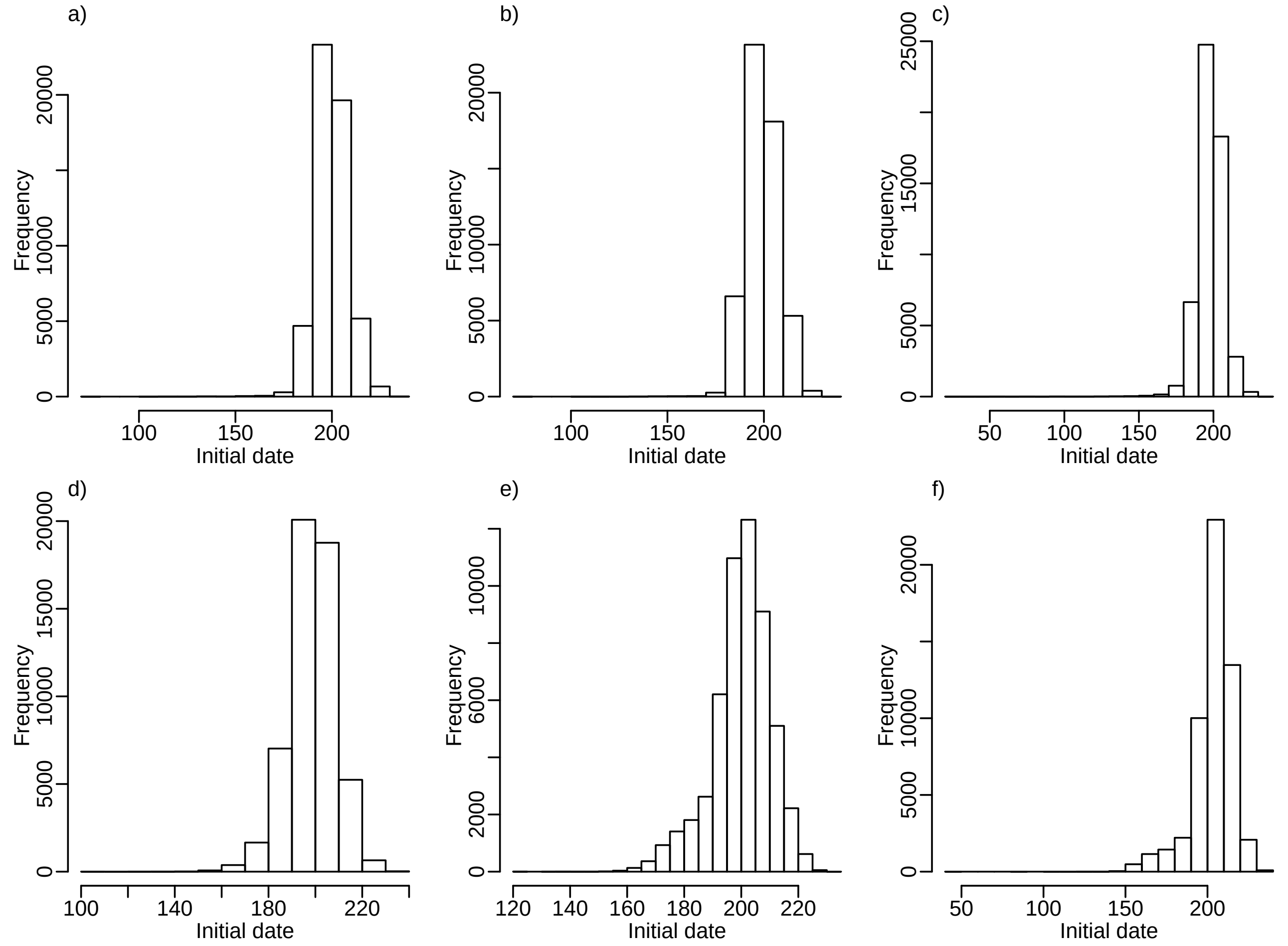

Figure S32: **Daily mobility seeding on 1st of April 2017**: Initial dates distribution over the different upazilas and 100 simulations for various seeding locations. a) 2017 simulation, b) Dhaka, c) Chittagong, d) Khulna, e) Rangpur f) Sylhet. Note that the scales on the y-axes depend on the seeding location.

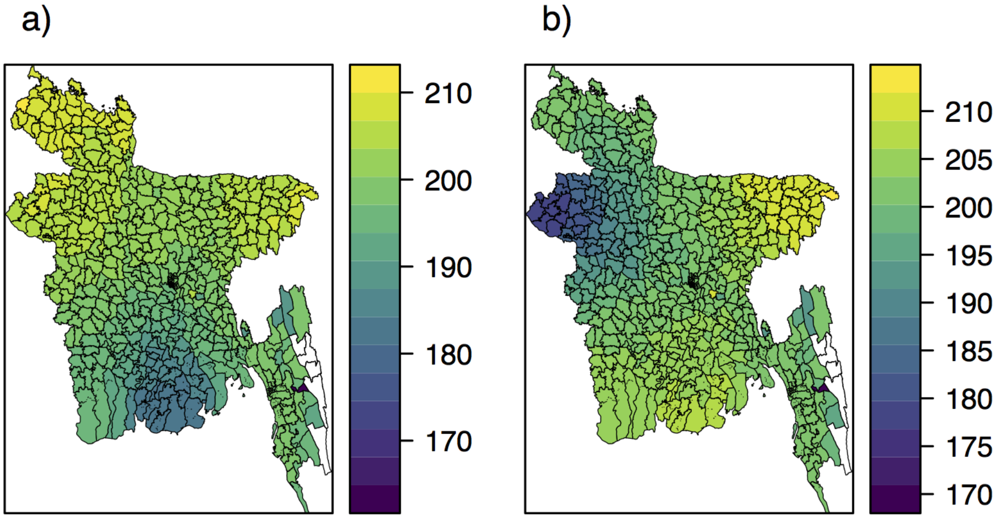

Figure S33: Mean initial dates per upazila when seeding in a) Barisal and b) Rajshahi. Note that the scales on the colour bars depend on the seeding location. 30th of September 2016 is defined as day 0.

than the other divisions, and the synchrony is similar to when seeding in Khulna, Rangpur, and Sylhet. When seeding in Barisal, the order of the initial dates in each division is Barisal, Khulna, Chittagong, Dhaka, Rajshahi, Sylhet, and Rangpur. When seeding in Rajshahi, the corresponding order is Rajshahi, Rangpur, Khulna, Chittagong, Dhaka, Barisal, and Sylhet. When seeding in Barisal, the mean difference in initial dates is slightly more than three weeks between Barisal and Rangpur. When seeding in Rajshahi, the mean difference in initial dates is also slightly more than three weeks between Rajshahi and Sylhet. The final sizes are the same as for the other seeding settings (0.228 (0.000172) when seeding in Barisal, 0.228 (0.000189) when seeding in Rajshahi). The global peak date and peak prevalence when seeding in Barisal are 272.1 (7.0) and 0.00716 ($2.21 \cdot 10^{-5}$). The global peak date and peak prevalence when seeding in Rajshahi are 272.4 (6.0) and 0.00715 ($2.31 \cdot 10^{-5}$). The global peaks are thus slightly delayed compared to seeding in Dhaka or Chittagong, but earlier than for the other seeding divisions. The peak prevalences are also slightly lower than when seeding in Dhaka and Chittagong. Standard deviations are given in parentheses.

S14 Gravity and radiation model

Plots of the estimated travel fluxes for the radiation model and the gravity model versus the time-averaged mobile phone mobility are given in Figure S37, along with the line $y = x$ for comparison. The radiation model clearly overestimates the travel volume on some links, while the gravity model does not appear to have this problem. The R^2 values are 0.31 for the gravity model and 0.65 for the radiation model. However, the gravity model estimates are closer to the $y = x$ line, and the estimates are thus closer with the gravity model than with the radiation model.

In addition, we perform a fluctuation analysis for the travel approximations, as is also done in Masucci et al. (2013) to compare the two models. We compute the Sørensen-Dice coefficient,

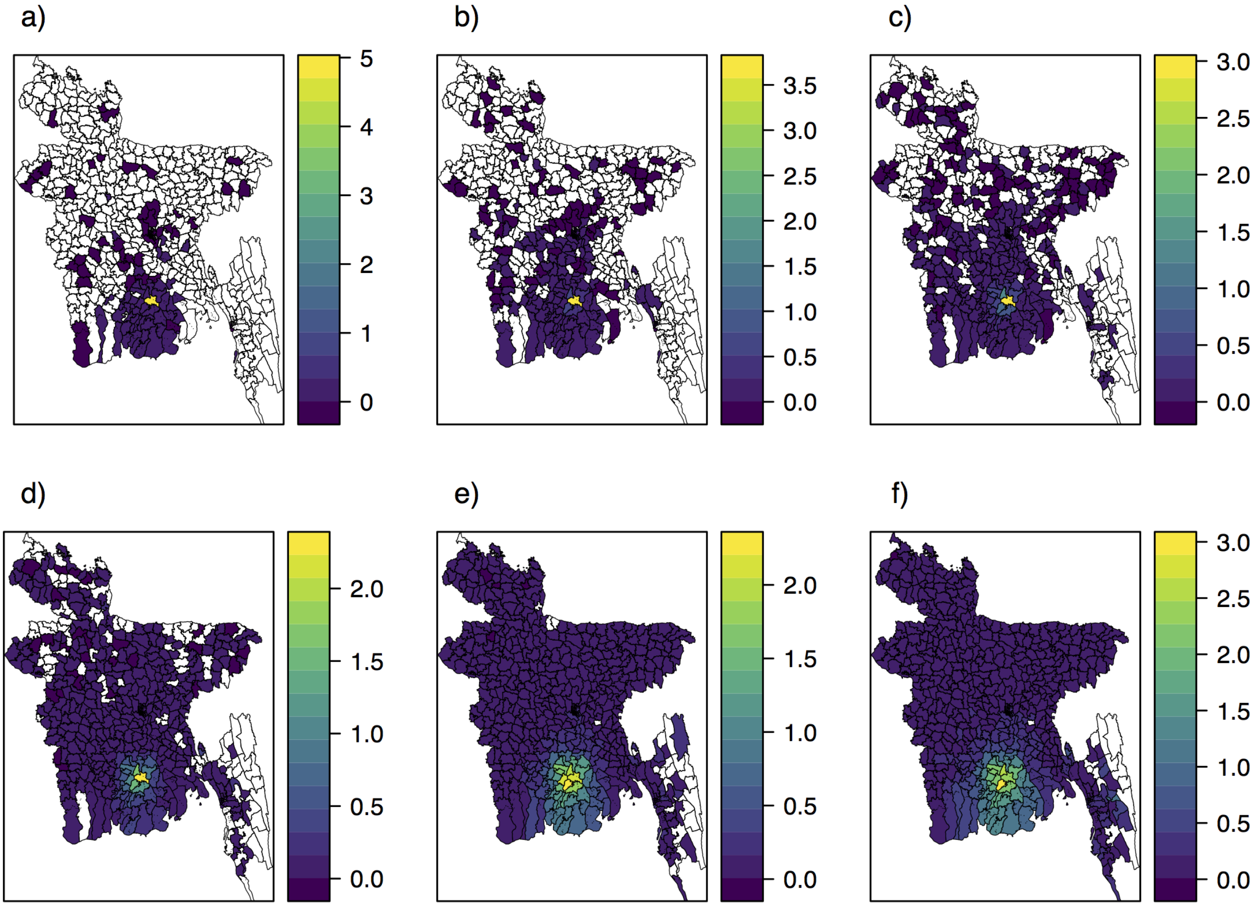

Figure S34: Mean prevalence per 100 000 residents in the different upazilas when seeding in Barisal, a) 2, b) 5, c) 10, d) 20, e) 40, and f) 50 days after the seeding event. Note that the scale is different for the different days.

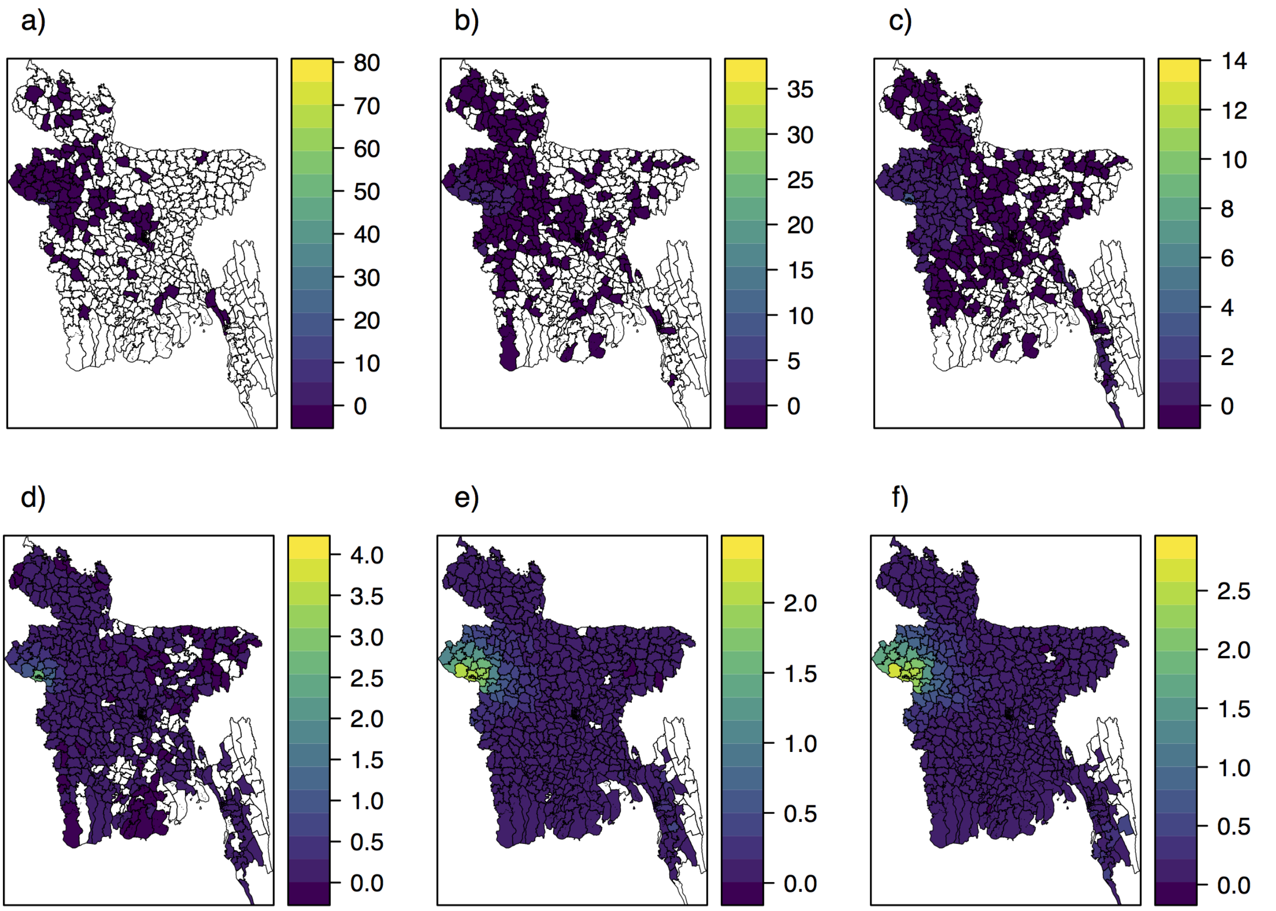

Figure S35: Mean prevalence per 100 000 residents in the different upazilas when seeding in Rajshahi, a) 2, b) 5, c) 10, d) 20, e) 40, and f) 50 days after the seeding event. Note that the scale is different for the different days.

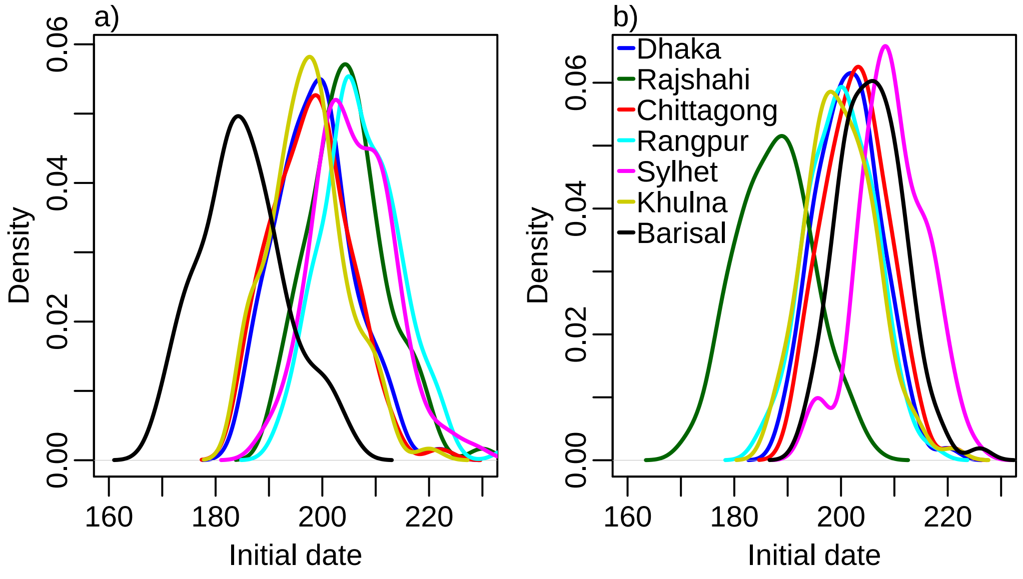

Figure S36: Initial dates distributions over the 100 simulations for a) Barisal and b) Rajshahi. 30th of September 2016 is defined as day 0.

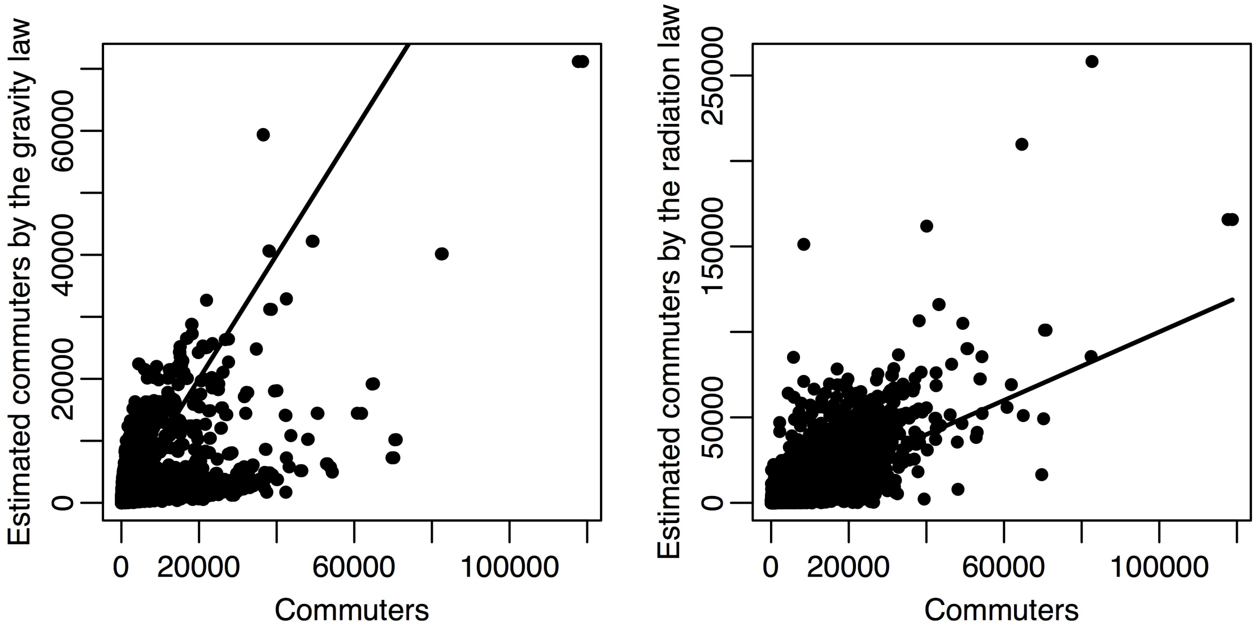

Figure S37: Travel fluxes versus the estimated data fluxes for the gravity model (left) and the radiation model (right). Note that the scales are not equal.

$E^{\text{Sørensen}}$, given as

$$E^{\text{Sørensen}} = \frac{2\sum_{i,j}\min(T_{ij}, w_{ij})}{\sum_{i,j} T_{ij} + \sum_{i,j} w_{ij}},$$

which is a similarity index between 0 and 1, where 0 means that there is no correlation, and 1 means that there is a perfect match. The $E^{\text{Sørensen}}$ is given in Figure S38, plotted in a distance and destination population size plane, and an origin population size and destination population size plane. We see that the gravity model performs the worst for large distances and small destination populations. The radiation model performs badly for short distances. The radiation model performs badly for travel between large and small origin and destination populations. The gravity model performs badly for small origin and destination population sizes. In addition, a plot showing which model performs the best in terms of $E^{\text{Sørensen}}$ is also given in Figure S38. The radiation model seems to overall perform better in terms of the Sørensen-Dice coefficient, except from for short distances, and travel between large and small populations, where the gravity model seems to perform better.

S15 Other influenza seasons

S15.1 Posterior distributions for 2014, 2015, and 2016

For the 2014-2016 influenza seasons, we use more informative priors based on the estimates for 2017, namely a normal distribution for β with mean 0.49 and standard deviation 0.02, and a uniform prior on [0, 0.001] on the reporting probability. The posteriors along with the priors are given in Figure S39. The posteriors clearly differ from the (informative) prior distributions based on the parameter estimates for the 2017 season. The posterior distributions for the transmission parameter are very informative about the parameter for each season, while the posterior distributions for the reporting probability are overlapping for the different seasons, indicating that we do not have much information about the reporting probability in our data.

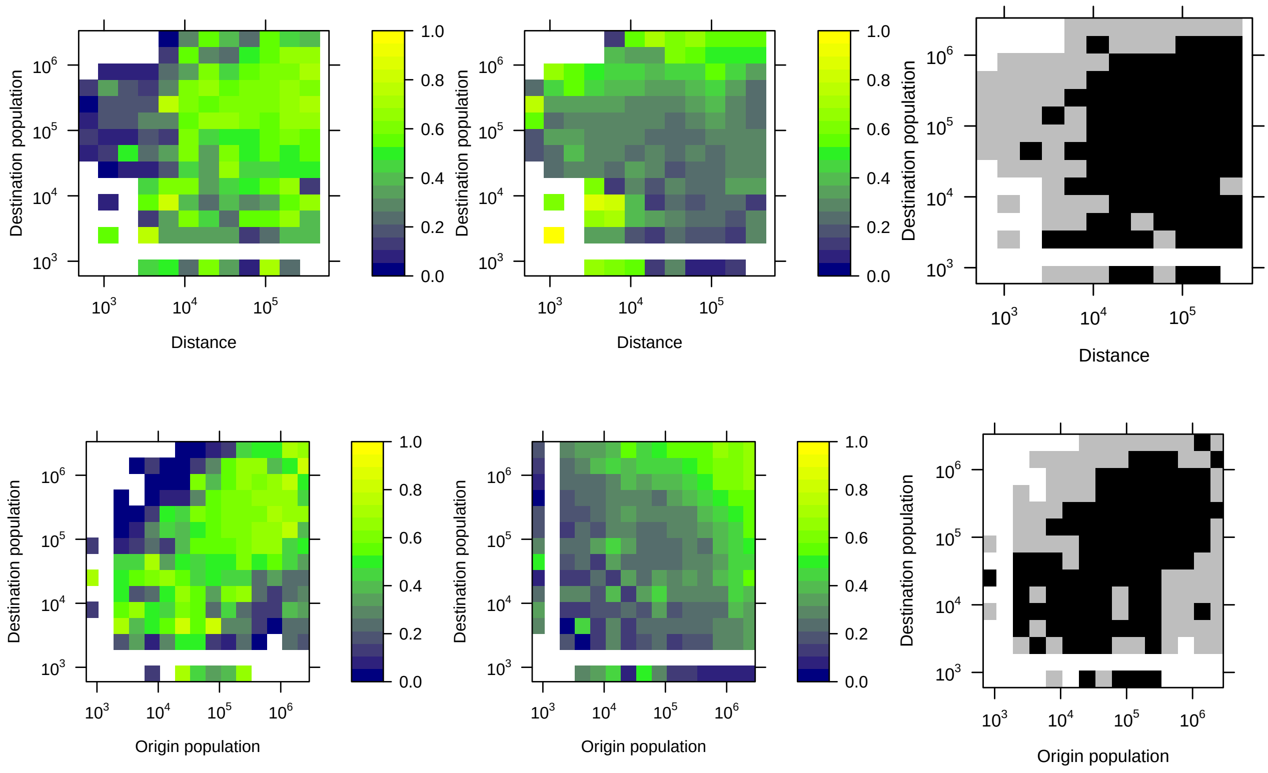

Figure S38: Sørensen-Dice coefficient in the distance and destination population plane (upper panel), and origin and destination population plane (lower panel) measured on log 10-scale, for the radiation model (left) and the gravity model (centre). To the left, an indicator of which mobility model performs the best in terms of the Sørensen-Dice coefficient is given, where black means that the radiation model outperforms the gravity model, and grey means that the gravity model outperforms the radiation model.

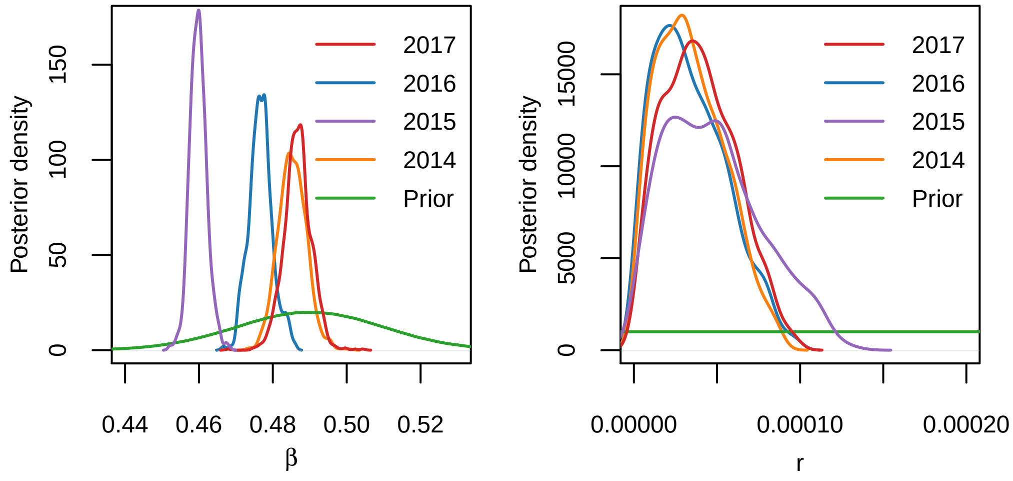

Figure S39: Posterior distributions for the different influenza seasons along with the informative prior used for the seasons 2014, 2015, and 2016.

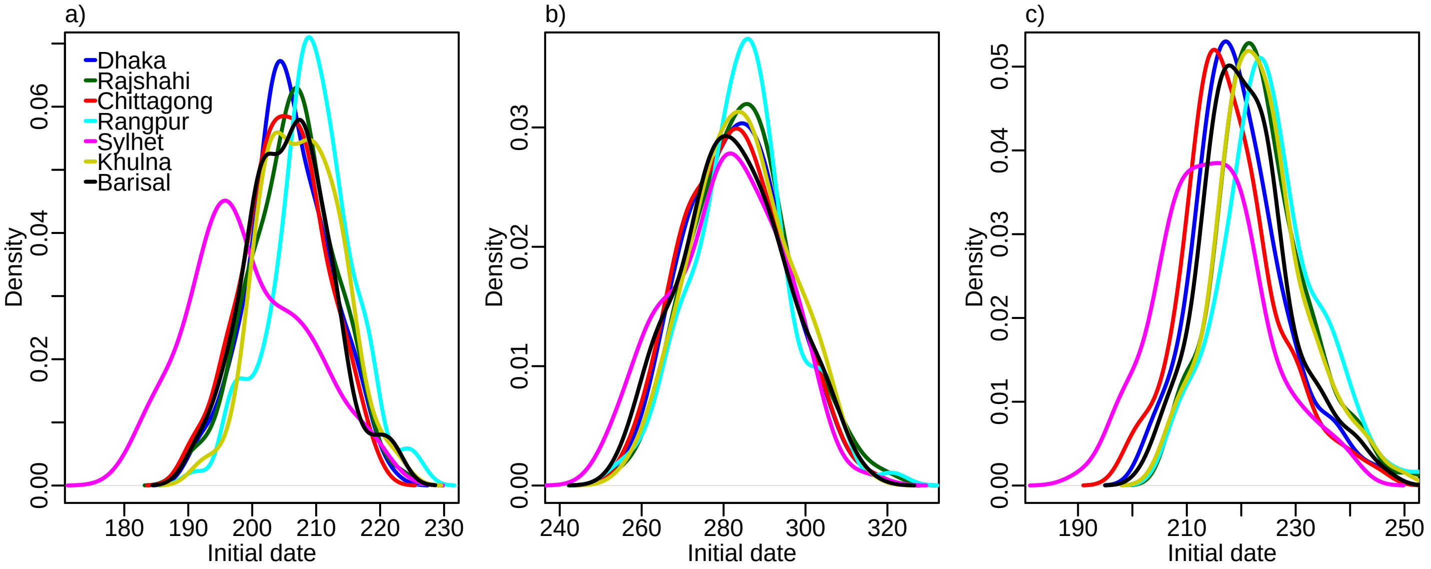

Figure S40: Initial dates distribution for each division, for different influenza seasons. a) 2016, b) 2015, and c) 2014.

S15.2 Simulations for 2014, 2015, and 2016

The resulting initial dates for each division for the 2014, 2015, and 2016 season simulations are given in Figure S40. For 2016, the epidemic seems to first spread in the Sylhet division. Considering the mean initial dates for the different divisions, the order of arrival is Sylhet, Chittagong, Dhaka, Barisal, Khulna, Rajshahi, and Rangpur. For 2015, the epidemic seems to be more synchronised across the different divisions. Considering the mean initial date for each division, the relative arrival times are Sylhet, Barisal, Chittagong, Dhaka, Khulna, Rangpur, and Rajshahi, in that order. Also for 2014, the epidemic seems to first spread in Sylhet. The order of arrival is Sylhet, Chittagong, Barisal, Dhaka, Rajshahi, Khulna, and Rangpur. Hence, the results are in agreement with our general results, where we find that Chittagong and Dhaka seem to be hit early in the epidemic, while the epidemics seem to have started in Sylhet. In recent years, our simulations thus indicate that there seems to have been an epidemic wave from East to West.

We plot the fitted incidence curves from our simulations, along with the case data for 2014, 2015, and 2016. The plot is given in Figure S41. The model fits the case data well for all the seasons. Note that we have plotted them to align the peak of the case data and the simulations as we have not estimated the initial conditions.

S16 Network descriptives and centrality measures

We compute various descriptive statistics of the time-averaged mobility network. The density of the network is the number of existing links divided by the number of possible links. The density of the time-averaged mobility network is 82%. The reciprocity of the network is the proportion of reciprocal links. The reciprocity is high–95% of the edges are reciprocal. The transitivity (proportion of triangles) is also high, 0.88. The diameter of the network is two, which means that no upazilas are more than two links away from each other. Hence, the mobility network is very dense and tightly connected.

We repeated the network analysis for the daily mobility matrices. The average density of the networks is 42% (standard deviation 3.1%), which is lower than for the time-averaged mobility matrix, as expected, but still a high density. The average reciprocity of the network is high, 79%

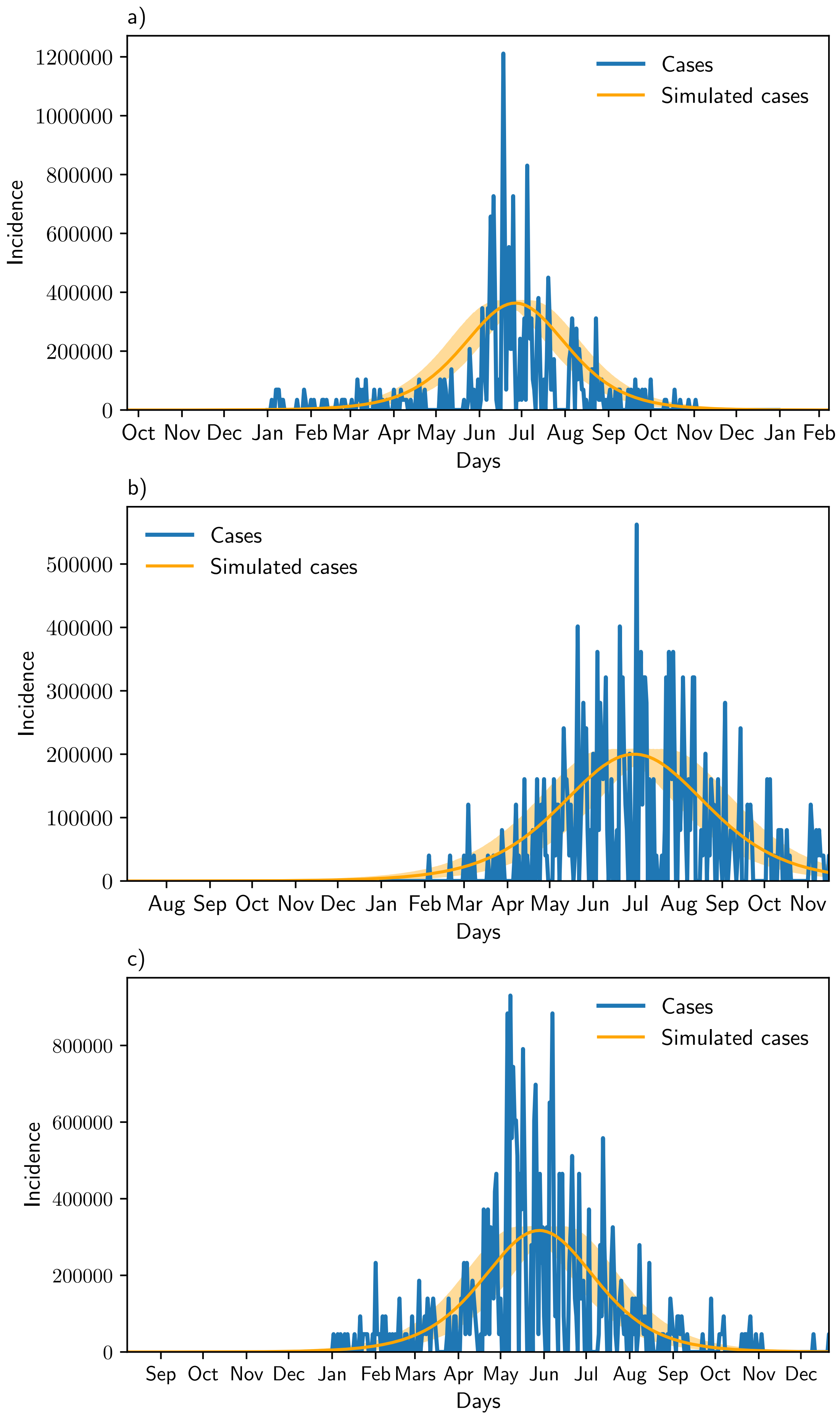

Figure S41: Mean incidence curve from simulations and scaled case data with estimated reporting probabilities for a) 2014, b) 2015, c) 2016, with 95% confidence interval.

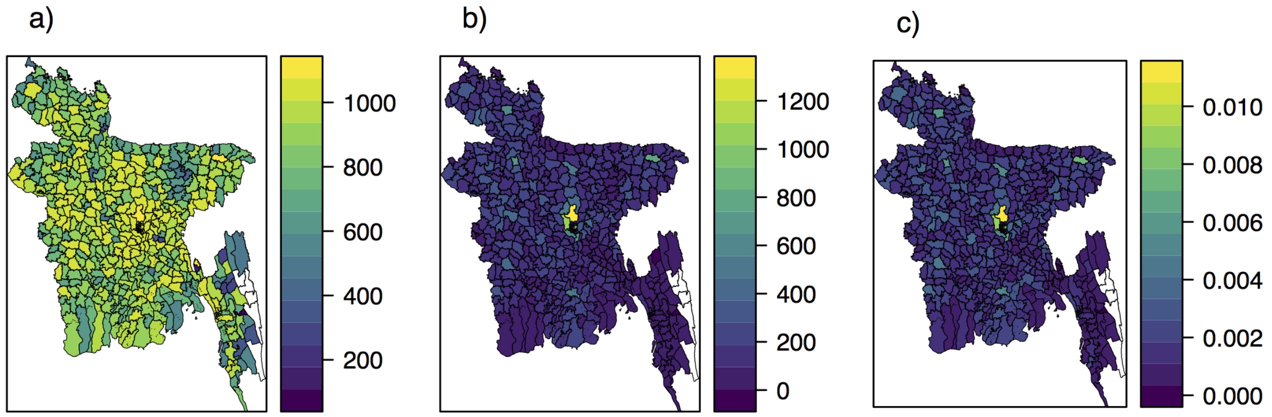

Figure S42: Centrality in each upazila for a) degree, b) strength (divided by 1000), and c) page rank centrality.

(standard deviation 1.6%), but lower than for the time-averaged mobility matrix. The average transitivity is 0.59 (standard deviation 0.023). The average diameter of the network is 2.01 (standard deviation 0.10). Hence, the daily mobility networks are dense and tightly connected.

We have computed the degree centrality, strength centrality, and page rank centrality for the different upazilas for the time-averaged mobility network, provided in Figure S42. Dhaka city seems central for all centrality indices, and the strength centrality is very similar to the page rank centrality. The upazilas with the highest degree are Badda (Dhaka), Jatrabari (Dhaka), Mirpur (Dhaka), Gazipur Sadar (Dhaka), Sylhet Sadar (Sylhet), which have the same degree. Gulshan (Dhaka), Kafrul (Dhaka), and Motijheel (Dhaka) have one degree lower, and Comilla Sadar Dakshin (Chittagong), Mohammadpur (Dhaka), Pallabi (Dhaka), Savar (Dhaka), Shahbagh (Dhaka), Uttara (Dhaka), Mymensingh Sadar (Dhaka), Sonargaon (Dhaka), Narayanganj Sadar (Dhaka), Narsingdi Sadar (Dhaka), Rangpur Sadar (Rangpur) have degree two lower than the maximum degree in the network. The ten upazilas with the highest strength are Gazipur Sadar (Dhaka), Gulshan (Dhaka), Savar (Dhaka), Badda (Dhaka), Mirpur (Dhaka), Sylhet Sadar (Sylhet), Narayanganj Sadar (Dhaka), Motijheel (Dhaka), Bogra Sadar (Rajshahi), and Mohammadpur (Dhaka), in descending order. The ten upazilas with the largest page rank centralities are Gazipursadar (Dhaka), Savar (Dhaka), Gulshan (Dhaka), Sylhet Sadar (Sylhet), Badda (Dhaka), Mirpur (Dhaka), Narayanganj Sadar (Dhaka), Bogra Sadar (Rajshahi), Motijheel (Dhaka), and Rangpur Sadar (Rangpur), in descending order.

Comparing with the network statistics for the radiation and gravity mobility networks, we find that the density for the radiation model, 0.51, is lower than for the time-averaged mobility network, while the density for the gravity network is almost 1 (0.9997). The diameter for the radiation network is 3, while the diameter for the gravity network is 2.

References

Bajardi, P., Poletto, C., Ramasco, J. J., Tizzoni, M., Colizza, V., and Vespignani, A. (2011). Human mobility networks, travel restrictions, and the global spread of 2009 H1N1 pandemic. *PLoS ONE*, 6(1):e16591.

Balcan, D., Colizza, V., Gonçalves, B., Hu, H., Ramasco, J. J., and Vespignani, A. (2009).

Multiscale mobility networks and the spatial spreading of infectious diseases. *Proceedings of the National Academy of Sciences of The United States of America*, 106(51):21484–21489.

Beaumont, M. A., Cornuet, J.-M., Marin, J.-M., and Robert, C. P. (2009). Adaptive approximate Bayesian computation. *Biometrika*, 96(4):983–990.

Birrell, P. J., Ketsetzis, G., Gay, N. J., Cooper, B. S., Presanis, A. M., Harris, R. J., Charlett, A., Zhang, X.-S., White, P. J., Pebody, R. G., and de Angelis, D. (2011). Bayesian modeling to unmask and predict influenza A/H1N1pdm dynamics in London. *Proceedings of the National Academy of Sciences of The United States of America*.

Birrell, P. J., Pebody, R. G., Charlett, A., Zhang, X.-S., and de Angelis, D. (2017). Real-time modelling of a pandemic influenza outbreak. *Health technology assessment (Winchester, England)*, 21(58):1.

Brooks-Pollock, E., Roberts, G. O., and Keeling, M. J. (2014). A dynamic model of bovine tuberculosis spread and control in Great Britain. *Nature*, 511(7508):228.

Colizza, V., Barrat, A., Barthelemy, M., Valleron, A.-J., and Vespignani, A. (2007). Modeling the worldwide spread of pandemic influenza: baseline case and containment interventions. *PLoS Medicine*, 4(1):e13.

Corbella, A., Zhang, X.-S., Birrell, P. J., Boddington, N., Pebody, R. G., Presanis, A. M., and de Angelis, D. (2018). Exploiting routinely collected severe case data to monitor and predict influenza outbreaks. *BMC public health*, 18(1):790.

Engebretsen, S., Engø-Monsen, K., Frigessi, A., and de Blasio, B. F. (2019). A theoretical single-parameter model for urbanisation to study infectious disease spread and interventions. *PLoS computational biology*, 15(3):e1006879.

Germann, T. C., Kadau, K., Longini, I. M., and Macken, C. A. (2006). Mitigation strategies for pandemic influenza in the United States. *Proceedings of the National Academy of Sciences of the United States of America*, 103(15):5935–5940.

Longini Jr, I. M., Halloran, M. E., Nizam, A., and Yang, Y. (2004). Containing pandemic influenza with antiviral agents. *American journal of epidemiology*, 159(7):623–633.

Masucci, A. P., Serras, J., Johansson, A., and Batty, M. (2013). Gravity versus radiation models: On the importance of scale and heterogeneity in commuting flows. *Physical Review E: Statistical, Nonlinear, and Soft Matter Physics*, 88(2).

Panigutti, C., Tizzoni, M., Bajardi, P., Smoreda, Z., and Colizza, V. (2017). Assessing the use of mobile phone data to describe recurrent mobility patterns in spatial epidemic models. *Royal Society open science*, 4(5):160950.

Presanis, A., Pebody, R., Paterson, B., Tom, B., Birrell, P., Charlett, A., Lipsitch, M., and de Angelis, D. (2011). Changes in severity of 2009 pandemic A/H1N1 influenza in England: a Bayesian evidence synthesis. *BMJ*, 343:d5408.

World Health Organization (2016). Influenza (seasonal). http://www.who.int/en/news-room/fact-sheets/detail/influenza-(seasonal). Accessed: 2016-08-15.
